# Photoresponsive Bilayer Coating Integrating a Porous Zinc Oxide Layer and a Chitosan–Antibiotic Drug Delivery System for Antimicrobial Photodynamic Therapy in Biomedical Implants

**DOI:** 10.1021/acsmaterialsau.6c00149

**Published:** 2026-10-08

**Authors:** Samuel S. Malheiros, Maria Helena R. P. Borges, Elidiane C. Rangel, Nilson C. Cruz, Carlos A. Fortulan, Xi Rao, Liqun Xu, João Gabriel S. Souza, Eduardo B. Moffa, Bruna E. Nagay, Valentim A. R. Barão

**Affiliations:** † Department of Prosthodontics and Periodontology Piracicaba Dental School, UniversidadeEstadual de Campinas (UNICAMP), Piracicaba, São Paulo 13566-590, Brazil; ‡ Laboratoryof Technological Plasmas, Institute of Science and Technology, SãoPaulo State University (UNESP), Sorocaba, São Paulo 18087-180, Brazil; § Department of Mechanical Engineering, University of São Paulo (USP), São Carlos, São Paulo 13566-590, Brazil; ∥ BRICS Joint Laboratory on Biomedical Materials School of Materials and Energy, Southwest University, Chongqing 400715, P. R. China; ⊥ Dental Research Division, Universidade Guarulhos, Guarulhos, São Paulo 07023-070, Brazil; # Faculdade Israelita de Ciências da Saúde Albert Einstein, Hospital Israelita Albert Einstein, SãoPaulo, São Paulo 05652-900, Brazil; ∇ Department of Dentistry, Centro Universitário das Faculdades Associadas (UNIFAE), São João daBoa Vista, São Paulo 13870-377, Brazil; ○ Department of Prosthodontics and Periodontology, Bauru School of Dentistry, University of SãoPaulo (USP), Bauru, São Paulo 17012-901, Brazil

**Keywords:** zinc, biomedical implant, plasma electrolytic oxidation, bioactive coatings, biofilms, antibiotic, photochemotherapy

## Abstract

Peri-implant infections remain a major cause of implant failure, highlighting the need for antimicrobial implant surfaces capable of suppressing biofilm formation while preserving the physicochemical and biological features needed for osseointegration. Here, we engineered a photoresponsive bilayer coating on titanium by anchoring a photosensitive antibiotic, demeclocycline (DMC), loaded into a polymeric chitosan film onto a zinc-doped porous oxide layer produced by plasma electrolytic oxidation (PEO). The inner PEO/Zn layer generated a porous ZnO-containing TiO_2_ matrix that provided mechanical interlocking for the polymeric film. The outer polymeric chitosan film deposition reduced surface roughness and hydrophilicity and enabled DMC incorporation. Chemical analyses confirmed the stepwise construction of the bilayer coating, revealing TiO_2_/ZnO porous oxide layer formation and chitosan and DMC incorporation. ROS generation under blue light was confirmed by the DCFH-DA fluorescence assay. Mechanically, the bilayer coating decreased the friction coefficient by approximately 75%, with the chitosan layer acting as a lubricating barrier under tribological stress. DMC release was sustained for 21 days with lysozyme-mediated degradation accelerating early drug release. Against saliva-derived polymicrobial biofilms formed for 96 h, the DMC-loaded coating reduced viable counts by approximately 2-log units under dark conditions, confirming intrinsic antibacterial activity. Upon blue light irradiation, the reduction reached approximately 3-log units, indicating on-demand photodynamic enhancement of the antimicrobial effect mediated by ROS. This response was accompanied by reduced metabolic activity, lower biofilm mass and matrix protein content, increased membrane-compromised cells assessed by live/dead, and suppression of more pathogenic microbial complexes. The coating also retained antibiofilm activity after microbial recolonization and the reusability of photodynamic therapy. Beyond antimicrobial activity, the coating preserved preosteoblastic cell viability and supported hydroxyapatite nucleation. Proteomic analysis showed an enriched immune, coagulation, protease-regulatory, and structural pathway proteomic profile. Overall, this bilayer platform integrates mechanical stability, sustained antibiotic delivery, and light-activated antimicrobial photodynamic therapy, representing a promising multifunctional coating for the control of implant infection.

## Graphical abstract

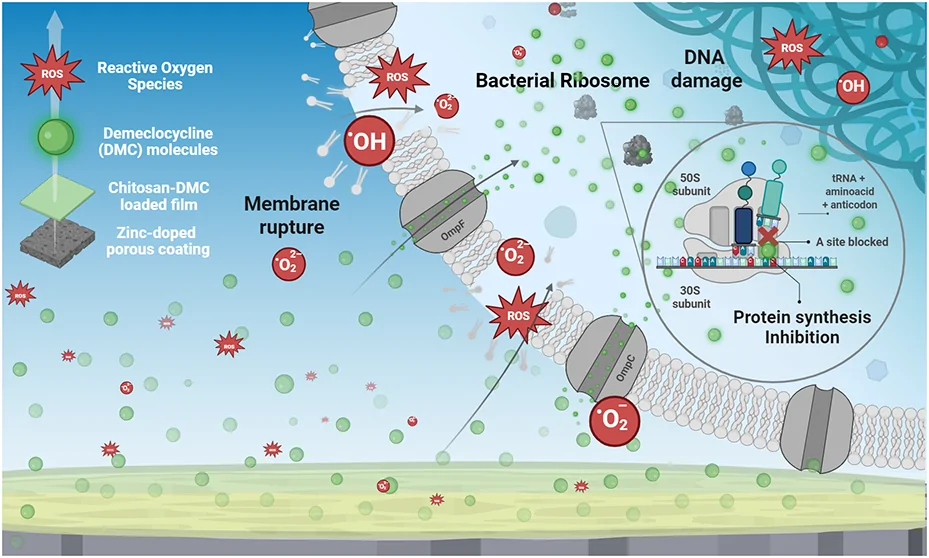

## Introduction

Peri-implant infections are a major biological complication in implant dentistry, compromising peri-implant tissue stability and the long-term survival of dental implants.^1^ These inflammatory diseases are mediated by polymicrobial biofilms and are characterized by inflammation of the peri-implant mucosa and progressive marginal bone loss.^2^ These conditions display a high prevalence, affecting 25 to 63% of patients with dental implants,^3^ although estimates can vary.^4,5^ Moreover, peri-implantitis may progress rapidly and cause extensive tissue destruction, ultimately leading to implant failure.^6,7^ This scenario is further aggravated by the expanding global dental implant market, which has surpassed several billion dollars annually and continues to grow,^8^ increasing the number of patients requiring the maintenance and management of biological complications. Despite its clinical relevance, no gold standard treatment has been established, and disease recurrence remains frequent after decontamination procedures.^9^

Among promising adjunctive therapies for peri-implant diseases, antimicrobial photodynamic therapy (aPDT) combines a photosensitizer, light, and molecular oxygen to generate reactive oxygen species (ROS).^10^ Upon irradiation at a wavelength matching the absorption spectrum of the photosensitizer, the excited molecules can trigger radical species and singlet oxygen capable of damaging multiple bacterial targets.^11^ Clinically, aPDT has been associated with improvements in clinical parameters^12,13^ and its localized action, broad antimicrobial spectrum, low invasiveness, and reduced potential for resistance selection support its use in peri-implant therapy.^14^ However, conventional photosensitizers act only during irradiation and provide limited residual protection against biofilm recolonization.^11^ Thus, a more effective strategy would be to use a photoresponsive molecule that combines sustained local antimicrobial activity with an on-demand photodynamic activation.

Demeclocycline (DMC), a tetracycline-class antibiotic, appears as an attractive alternative to conventional photosensitizers because it combines intrinsic bacteriostatic activity with photoresponsive antimicrobial potential.^15^ Beyond its classical antibiotic mechanism of inhibiting bacterial protein synthesis, DMC-conjugated tetracyclic structure enables absorption and photochemical activation under UVA and blue light, leading to ROS generation and antimicrobial activity against Gram-positive and Gram-negative bacteria, including resistant strains.^11,16,17^ This feature is particularly relevant because, unlike UV irradiation, which is associated with carcinogenic risks,^18^ blue light offers a safer and already clinically employed wavelength.^19^ Unlike conventional photosensitizers, DMC can sustain an antimicrobial effect beyond the irradiation period;^20^ however, free DMC may rapidly diffuse from the implant interface, limiting local retention and therapeutic duration.^21^ Therefore, one of the novelties of the present strategy lies in integrating DMC into an implant coating that preserves its dual antibiotic/photosensitizer function while enabling local retention, controlled release, and on-demand photodynamic activation.

Chitosan (CS), the deacetylated derivative of chitin, is a particularly attractive polymeric platform for interfacial drug delivery because it is biodegradable, biocompatible, and structurally tunable.^22^ Its hydroxyl and amino groups enable electrostatic interactions and hydrogen bonding, supporting the formation of functional films capable of incorporating therapeutic agents.^23^ In implant-related applications, CS-based coatings have already been explored as drug delivery matrices because they can protect, modulate, and sustain drug release.^24,25^ These properties make CS suitable for retaining DMC at the implant interface, providing sustained local antibiotic availability, while preserving light-triggered photodynamic activation. However, polymeric coatings on titanium remain vulnerable to weak interfacial adhesion and delamination under mechanical loading.^24,26^ Moreover, because polymer degradation progressively exposes the underlying surface,^27^ a mechanically stable anchoring substrate is required to retain the film and preserve a favorable interface for peri-implant tissue integration.

Plasma electrolytic oxidation (PEO) alongside approaches such as anodization, hydrothermal treatment, and chemical functionalization used to generate nanotubular or bioactive titanium surfaces^28,29^ provides a suitable anchoring strategy. By converting titanium into a highly adherent porous oxide layer, PEO enables the simultaneous incorporation of bioactive ions.^30,31^ Zinc (Zn) is particularly relevant because it combines antimicrobial activity with biological effects associated with protein adsorption, osteoblast function, and mineralized tissue formation.^32,33^ Previously, our group developed a Zn-doped PEO coating composed of a ZnO/TiO_2_ matrix that improved crystallinity, electrochemical and tribological resistance, protein adsorption, hydroxyapatite formation, and ex vivo implant stability while reducing biofilm accumulation.^34^ Therefore, this zinc-doped porous PEO coating represents a rational anchoring platform for a DMC-loaded CS film, as it can interlock the polymeric layer while providing a bioactive and highly resistant substrate capable of maintaining long-term functionality after progressive polymer degradation.

Here, we developed a photoresponsive bilayer coating by anchoring a DMC-loaded CS film onto a zinc-doped porous PEO layer on titanium. This architecture integrates the mechanical stability and bioactivity of the PEO/Zn substrate with the drug-retention capacity of CS and the dual antibiotic–photosensitizer function of DMC. The surface modification strategy is summarized in Figure 1. The bilayer interface supported DMC incorporation and sustained release and provided enhanced antibiofilm activity against saliva-derived polymicrobial biofilms. DMC-loaded coatings reduced biofilm viability, metabolic activity, extracellular matrix accumulation, and peri-implantopathogenic microbial complexes, while blue-light irradiation further potentiated the antimicrobial response and maintained activity after recolonization. Beyond infection control, the coating preserved pre-osteoblastic cell viability, promoted hydroxyapatite formation, and maintained a biologically favorable protein adsorption profile, supporting its potential as a multifunctional implant surface for peri-implant infection prevention and control.

**Figure 1:**
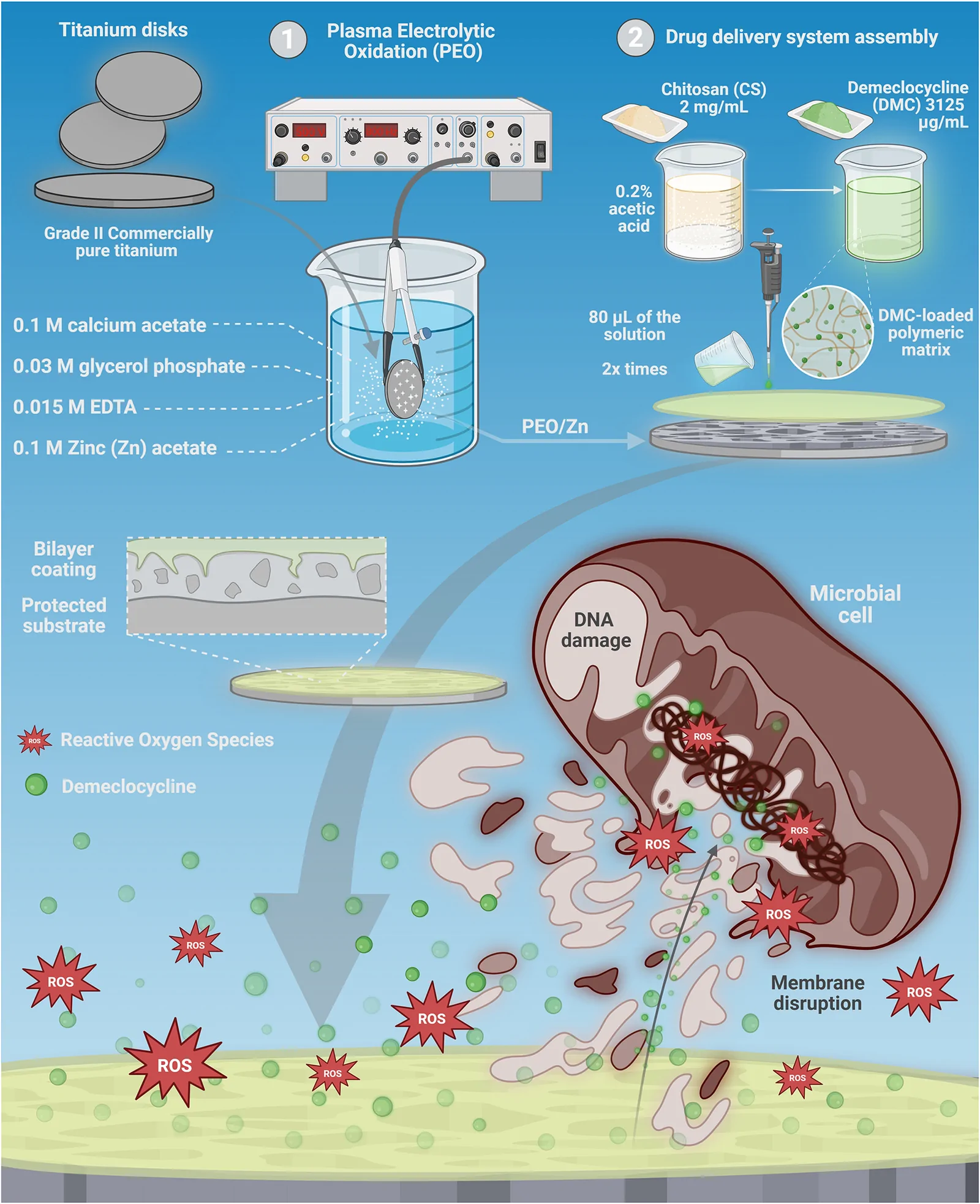
Schematic overview of the photoresponsive bilayer coating assembly integrating a zinc-doped porous PEO anchoring layer with a demeclocycline loaded chitosan film for sustained and light activated antimicrobial effect on titanium implant surfaces. Created in BioRender.

## Results and Discussion

### Coatings Morphology

The scanning electron microscopy micrographs (Figure 2A) revealed distinct morphological characteristics among the control and experimental groups. The Ti group exhibited a polished surface with visible longitudinal grooves, which is a feature characteristic of mechanical polishing.^35^ In contrast, the PEO-treated groups displayed a porous morphology, which is related to the dielectric breakdown mechanism occurring during PEO, where the applied high-voltage electric fields exceed the oxide layer’s dielectric strength, leading to localized microdischarges. With repeated plasma events, material ejection from discharge channels and rapid solidification result in a porous architecture.^36^ Compared with PEO, PEO/Zn surfaces exhibited a more compact and homogeneous microstructure with smaller pores, in agreement with a previous report.^34^ This effect is likely related to the increased electrolyte conductivity promoted by zinc acetate dissociation, which favors less intense but more frequent microdischarges, resulting in smaller and more uniformly distributed pores.^34^ Subsequent deposition of the CS-based film further modified the surface features of PEO/Zn@CS and PEO/Zn@CS/DMC. SEM micrographs confirmed a low-contrast polymeric layer partially covering the PEO/Zn surface, while the underlying pores remained discernible, suggesting that the film integrated and conformed to the preexisting surface. The incorporation of DMC into the CS film did not visibly alter the morphology.

**Figure 2:**
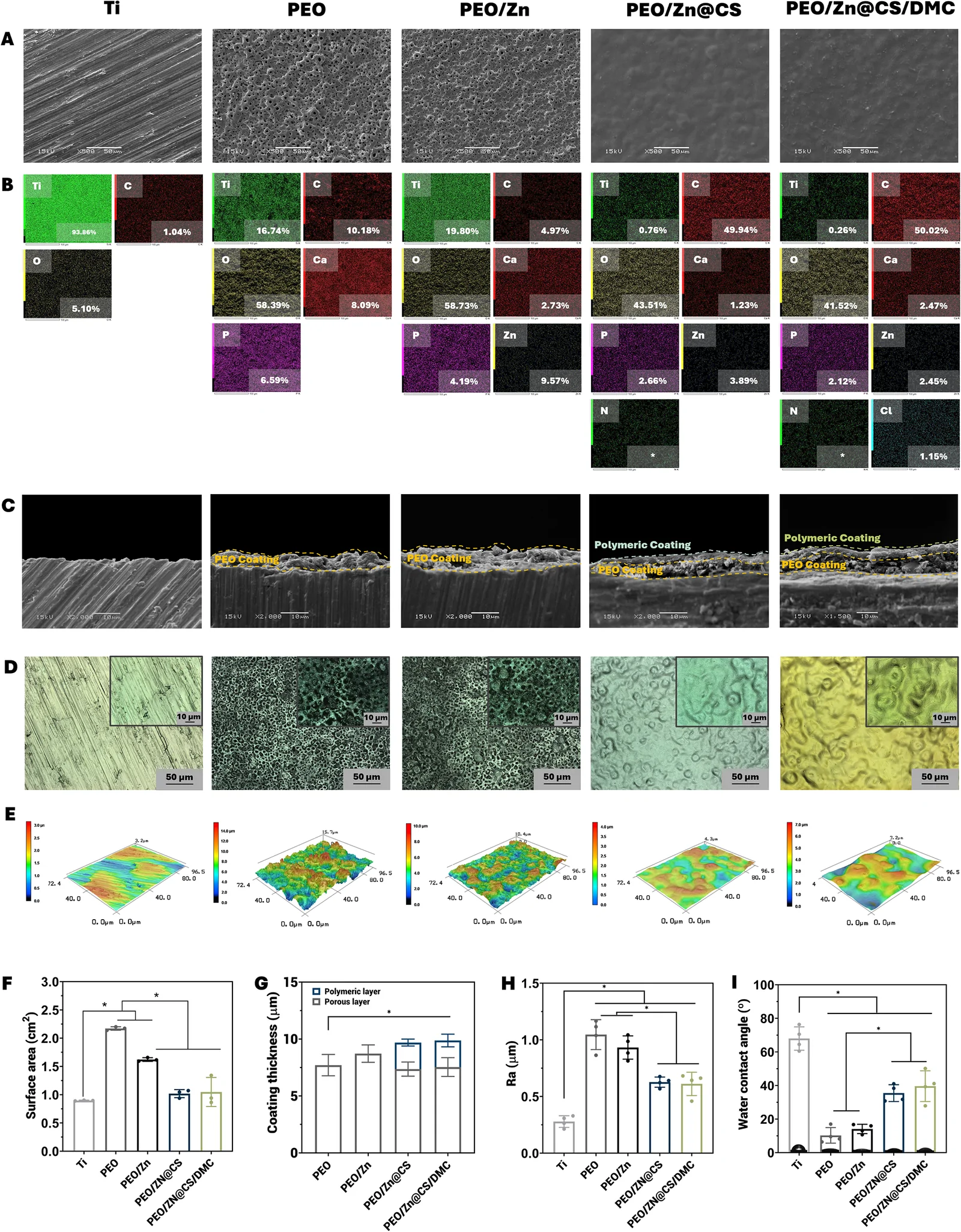
Morphological and topographical characterization of control and PEO-treated surfaces. (A) SEM micrographs showing top-view surface morphologies of Ti, PEO, PEO/Zn, PEO/Zn@CS, and PEO/Zn@CS/DMC groups (15 kV, magnification 500×, scale bar = 50 μm). (B) EDS color mapping indicating the spatial distribution of elements and their corresponding atomic percentages across the coatings. (C) Cross-sectional SEM micrographs depicting the bilayer structure of the coatings (15 kV, magnification 2000×, scale bar = 10 μm). (D) Confocal optical and laser scanning microscopy (CLSM) merged images at 50× and 150× magnification (scale bars = 10 μm and 50 μm, respectively). (E) Three-dimensional projections of the surface topography obtained via CLSM. (F) Quantitative surface area measurements (cm^2^) derived from CLSM analysis. (G) Coating thickness (μm) measurements. (H) Average surface roughness (Ra, μm). (I) Water contact angle (°) measurements assessing surface wettability. Data are expressed as mean ± standard deviation. Statistically significant differences between groups are indicated by symbols, with * representing *p* < 0.05, as determined by one-way ANOVA with Tukey’s post hoc test for multiple comparisons.

Energy-dispersive spectroscopy (EDS) elemental mapping (Figure 2B) confirmed a homogeneous distribution of intrinsic and extrinsic incorporated elements across all experimental surfaces. Also, cross-sectional SEM micrographs (Figure 2C) corroborate the hierarchical structure formed by each treatment. While the Ti group lacked a coating, the PEO and PEO/Zn groups exhibited an oxide-porous layer. The presence of the polymeric film in PEO/Zn@CS and PEO/Zn@CS/DMC groups was evident as an additional layer above the PEO-treated surfaces. Notably, the polymeric film appeared to infiltrate the porous network, suggesting a mechanical interlocking between the CS layer and the underlying porous coating. This interfacial integration is particularly advantageous, since it can enhance the adhesion of the polymeric layer, which is often a challenge on smoother surfaces.^30^ In the absence of an anchoring layer, polymer coatings frequently require chemical adhesion promoters or crosslinking agents,^37^ which may complicate large-scale industrial production and may introduce cytocompatibility concerns. Therefore, the porous biocompatible nature of the PEO layer facilitated superior anchorage of the CS coating, potentially improving its durability and wear resistance, as previously reported for other polymeric coatings associated with PEO surfaces.^30^

Confocal microscopy images provided additional insights into surface topography, reinforcing SEM findings. Both 50× and 150× magnifications (Figure 2D) demonstrated the characteristic porous structure of PEO-treated surfaces, while also revealing the adaptation of the CS-based film to the underlying architecture. Three-dimensional reconstructions (Figure 2E) further confirmed that the polymeric film reduced vertical surface discrepancies, as regions initially displaying high roughness in PEO-treated samples appeared more leveled after polymer deposition. This was particularly evident in confocal surface maps, where high-intensity red and orange regions (representing peaks) were less prominent in the PEO/Zn@CS and PEO/Zn@CS/DMC groups, while dark blue valleys (indicating deeper surface recesses) appeared less pronounced. A previous study showed that multilayered polymeric coatings offer a key advantage by preserving the substrate’s morphological features depending on the number of layers.^24^ Since in this study we used only two CS layers, porous topography was maintained, which is a particularly relevant feature for dental and orthopedic implants, where micro topographical features can influence osteoblast adhesion, and consequently osseointegration.^38^

Surface area analysis (Figure 2F) quantitatively reflected these topographical modifications. The polished Ti group exhibited a surface area of 0.90 cm^2^, whereas PEO treatment significantly increased this value to approximately 2.2 cm^2^ (*p* = 0.0001). The incorporation of zinc into the PEO process resulted in a more compact microstructure, reducing the surface area to 1.60 cm^2^, consistent with SEM and confocal images. The deposition of the polymeric film further decreased the surface area, with both PEO/Zn@CS and PEO/Zn@CS/DMC groups exhibiting statistically significant reductions relative to the PEO group (*p* < 0.05), reaching values closer to 1.0 cm^2^. This reduction is likely attributable to the partial filling of pores and smoothing of surface irregularities by the polymeric coating. Both PEO and PEO/Zn groups exhibited coating thicknesses of approximately 8 μm (Figure 2G). In the PEO/Zn@CS and PEO/Zn@CS/DMC groups, a slight but statistically significant increase in total coating thickness was observed compared to PEO alone (*p* < 0.05). This increase is primarily attributed to the polymeric layer covering surface irregularities and infiltrating porosities, rather than forming a thick, distinct layer. Yet, direct measurements confirmed that the CS-based coating with or without DMC exhibited an approximate thickness of 2 μm.

Mean roughness (Ra) assessed by profilometer followed a similar trend (Figure 2H). The Ti group displayed a low Ra, characteristic of polished surfaces, while PEO treatment induced a significant increase (*p* < 0.001) due to the formation of microscale surface features.^31^ No significant differences were observed between PEO and PEO/Zn groups (*p* > 0.05), indicating that Zn incorporation did not markedly alter roughness parameters. Following CS film deposition, Ra values significantly decreased (∼0.6 μm, *p* < 0.003), supporting the hypothesis that polymer infiltration within surface pores contributed to the observed smoothing effect. Another critical factor influencing interactions between organic and inorganic materials is surface wettability, which was also modified by the treatments (Figure 2I). While the Ti group exhibited a water contact angle of approximately 70°, both PEO and PEO/Zn groups demonstrated a significant decrease (*p* < 0.0001), indicative of enhanced hydrophilicity. This effect is likely attributed to surface hydroxylation during PEO, since the formation of hydroxyl radicals and functional groups containing oxygen can increase the substrate’s affinity for aqueous environments.^39^ The PEO/Zn@CS and PEO/Zn@CS/DMC groups exhibited a slight increase in water contact angle compared to PEO treated surfaces, which can be related to compositional differences introduced by the polymeric film, as CS is rich in carbon and nitrogen, which modulate surface polarity and hydrophilic interactions.^40^ Nonetheless, despite this moderate increase, surfaces coated with CS remained significantly more hydrophilic than polished Ti, which may favor initial interactions with biological fluids relevant to the early tissue response.^41^

### Chemical and Optical Characterization

Semi-quantitative EDS analysis provided detailed elemental composition of the control and experimental coatings (Figure 3A). The untreated Ti group primarily exhibited titanium, oxygen, and carbon at quantifiable levels, with oxygen likely originating from native oxide layers that are naturally formed on the Ti surface and carbon likely from environmental contamination adsorbed onto the material.^39^ Following PEO treatment, a substantial increase in the atomic percentage of oxygen was observed, corroborating the formation of the oxide coating induced by plasma-assisted oxidation.^36^ Moreover, Ca and P were effectively incorporated into the surface, with atomic percentages of 8.1 and 6.6 atom %, respectively. This incorporation is expected, given the presence of calcium acetate and glycerol phosphate in the electrolyte solution, which, after dissociation, facilitate Ca^2+^ and PO_4_^3–^ diffusion into the growing oxide matrix under the high-energy conditions of PEO.^42^

**Figure 3:**
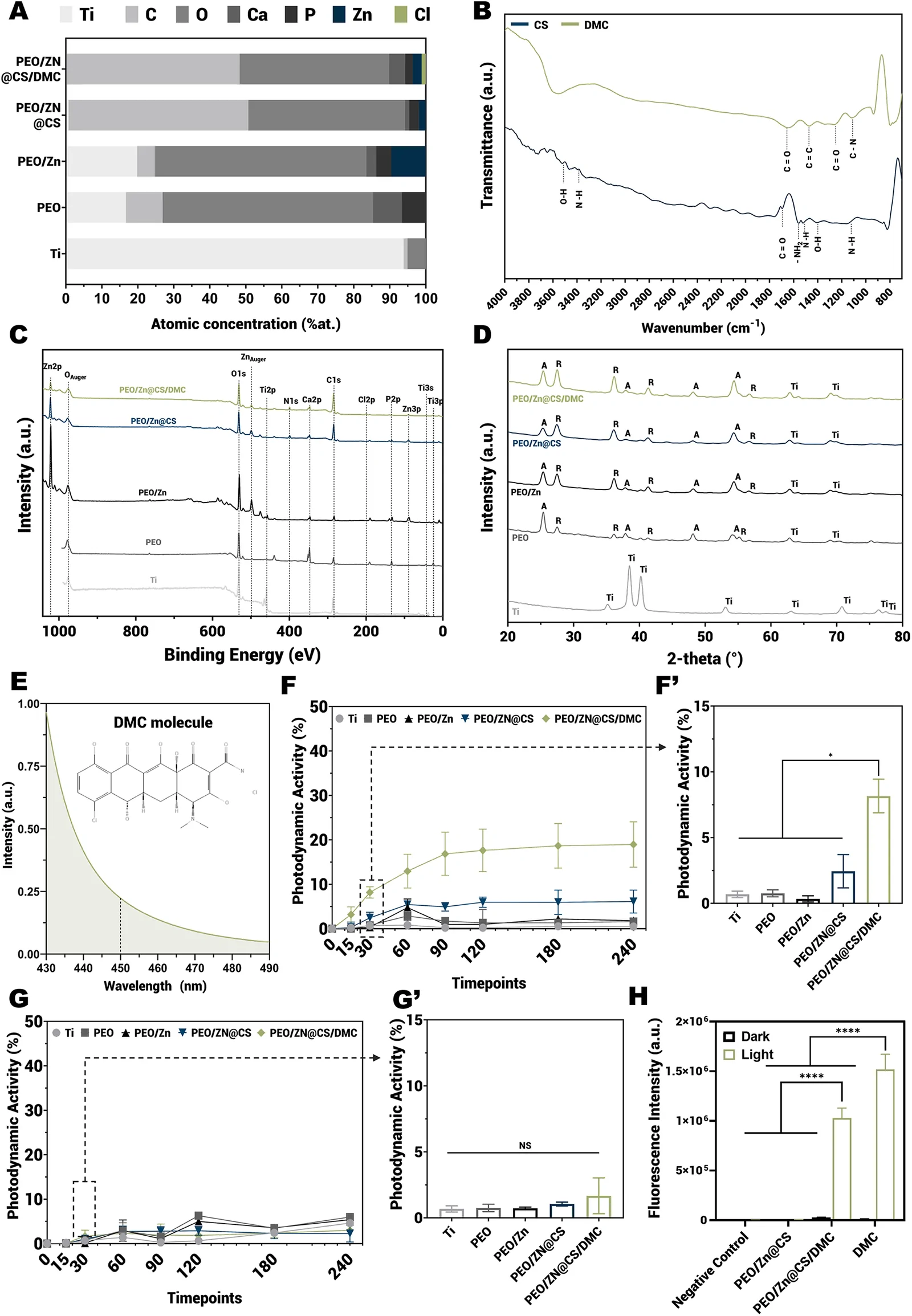
Chemical characterization of control and experimental coatings. (A) Elemental composition analysis displaying atomic elemental percentage distribution determined by energy-dispersive X-ray spectroscopy (EDS). (B) Fourier-transform infrared spectroscopy (FTIR) spectra of PEO/Zn@CS and PEO/Zn@CS/DMC coatings. (C) X-ray photoelectron spectroscopy (XPS) full spectra for all groups, with dotted lines indicating key binding energy peaks associated with surface elemental composition. (D) X-ray diffraction (XRD) patterns displaying characteristic diffraction peaks corresponding to crystalline phases. (E) Ultraviolet-visible (UV-Vis) absorption spectra of demeclocycline solution at 500 μg/mL. (F) Photodynamic activity of Ti, PEO, PEO/Zn, PEO/Zn@CS, and PEO/Zn@CS/DMC coatings under blue-light irradiation over 240 min. (F’) Quantitative analysis of photodynamic activity after 30 min of light exposure. (G) Photodynamic potential of all groups under dark conditions, with no light activation. (G’) Comparative analysis of photodynamic activity in dark conditions after 30 min. (H) Reactive oxygen species (ROS) generation assessed by the DCFH-DA fluorescence assay under light and dark conditions. Data are expressed as mean ± standard deviation. Statistically significant differences between groups are indicated by symbols, with * representing *p* < 0.05, as determined by one-way ANOVA with Tukey’s post hoc test for multiple comparisons. Illustration created in BioRender (License number: MV27W676D5).

In the PEO/Zn group, the addition of zinc acetate to the electrolyte resulted in the successful incorporation of Zn into the coating, reaching 9.6 atom %. However, this modification also resulted in a reduction in Ca (2.7 atom %) and P (4.2 atom %) content, suggesting a competitive incorporation mechanism during PEO processing.^43^ This competition happens because multiple charged ionic species coexist during plasma discharges, leading to interactions between metal cations and anionic complexes at the electrode electrolyte interface.^44^ A key factor that increased Zn incorporation is the presence of EDTA in the electrolyte solution. EDTA serves as a chelating agent, forming a negatively charged Zn EDTA complex, which can facilitate electromigration of anionic species toward the substrate.^34^ This process enables Zn incorporation through both electromigration and diffusion mechanisms that act synergistically to enhance the coating’s zinc content. The anionic Zn-EDTA complex competes with phosphate ions (PO_4_^3–^) for integration into the oxide lattice, leading to the observed reduction in phosphorus content. Additionally, free Zn^2+^ ions compete with Ca^2+^ for incorporation, further explaining the reduction in calcium levels in the PEO/Zn group compared to PEO alone.^34^

Following polymeric film deposition, the elemental profile underwent significant changes. In the PEO/Zn@CS and PEO/Zn@CS/DMC groups, the Ti substrate was nearly undetectable with atomic percentages below 1%, indicating that the polymeric coating shielded the underlying metal surface from EDS detection. Moreover, the application of the polymeric film also led to a decrease in Ca, P, and Zn signals relative to those of the uncoated PEO/Zn group. This reduction is an expected artifact of semi-quantitative EDS analysis, in which atomic percentages are normalized to the detected elements. Because the polymeric layer introduced a dominant carbon and oxygen signal, the relative percentages of the underlying elements (Ca, P, and Zn) decreased, even though these elements remained incorporated within the oxide layer. This phenomenon is expected since the electron beam may not have penetrated sufficiently through the bilayer coating. As a result, the strong signal from the overlying polymeric layer dominated the spectra.

The polymeric film led to a substantial increase in carbon content, with atomic percentages approaching 50% in both PEO/Zn@CS and PEO/Zn@CS/DMC groups. This sharp increase is directly attributable to the composition of the CS and DMC. The partial deacetylation of chitin to obtain CS promotes the formation of repeating glucosamine (C_6_H_11_NO_4_) and N-acetylglucosamine (C_8_H_15_NO_6_) units.^45^ The presence of carbonyl and hydroxyl (−OH) functional groups in the polymer structure contributes to the elevated carbon and oxygen signals detected in EDS spectra.^24^ Additionally, the DMC molecule, incorporated into the PEO/Zn@CS/DMC group, also contributed to the compositional profile of the coating. DMC is characterized by multiple carbon and oxygen-containing functional groups, including hydroxyl (−OH), amide (−CONH_2_), and ketone (C = O) moieties, which also contributed to the increase in these elements’ presence. Additionally, the molecular structure of DMC hydrochloride (C_21_H_21_ClN_2_O_8_·HCl) includes chlorine (Cl), as detected in EDS mapping. The chlorine signal appeared exclusively in the antibiotic-loaded coating, reinforcing that DMC successfully integrated into the polymeric layer without significant loss during deposition. Interestingly, EDS mapping confirmed the presence of nitrogen in the polymeric-coated groups; however, quantitative EDS analysis could not accurately measure nitrogen levels because the detected signal fell below the equipment’s quantification limit. The inability to precisely quantify nitrogen may stem from the relatively low atomic weight of nitrogen and potential interference from background signals.^46^ Nonetheless, the presence of CS- associated amine groups (−NH_2_) and demeclocycline-derived nitrogen moieties was qualitatively confirmed through spectral mapping and chemical bonding analysis.

Fourier Transformed InfraRed (FTIR) analysis was performed to identify the functional groups present in the polymeric film, confirming the incorporation of CS and demeclocycline into the coating (Figure 3B). In the PEO/Zn@CS group, a peak was observed around 3500 cm^–1^, corresponding to the O–H stretching vibrations of hydroxyl groups, which are abundantly present in the glucosamine units of CS.^40^ This band overlapped with another stretching vibration at ∼3400 cm^–1^, which is attributed to N–H stretching from primary (−NH_2_) and secondary (−NH) amine groups. These signals confirm the preservation of amine-rich functional groups, which are fundamental for CS’s bioactivity.^27^ Another peak was detected at ∼1700 cm^–1^, corresponding to the C=O stretching vibration of carbonyl groups. This signal is most likely associated with residual N-acetyl groups (−NHCOCH_3_) from partially deacetylated chitin, leaving a fraction of its polymeric backbone in N-acetylglucosamine form.^47^ Further confirmation of CS’s characteristic chemical structure was observed in the spectral region between 1500–1600 cm^–1^, where overlapping N–H bending vibrations and O–H deformations suggest strong hydrogen bonding interactions within the polymeric film.^40^ The persistence of these functional groups is essential for CS’s ability to act as a drug-delivery system for DMC.

The FTIR spectrum of the PEO/Zn@CS/DMC group exhibited new absorption bands that confirmed the incorporation of DMC in the polymeric film. A strong C=O stretching vibration at ∼1650 cm^–1^ was detected, which is characteristic of ketone and amide functional groups present in tetracycline-class molecules.^48^ This absorption is significant, as tetracycline antibiotics rely on their functional groups for metal chelation and interaction with bacterial ribosomal subunits, which are central to their antimicrobial mechanism of action.^20^ The presence of this functional group in the coating suggests that demeclocycline maintained its structural integrity during incorporation into the polymeric matrix. Another peak around 1500 cm^–1^ was assigned to C=C stretching vibrations, confirming the presence of conjugated aromatic systems^48^ present in demeclocycline’s molecular structure. The C–N stretching vibration at ∼1100 cm^–1^ also appeared in the PEO/Zn@CS/DMC group, indicating that the amide and tertiary amine groups characteristic of tetracyclines were retained after drug incorporation.^48^

X-ray photoelectron spectroscopy (XPS) provided an in-depth assessment of the elemental composition and electronic states within the experimental coatings. The full-spectrum survey (Figure 3C) captured photoemission signals corresponding to the core levels of the elements present in each sample. For the Ti control group, the XPS spectra exhibited characteristic peaks corresponding to Ti 2p (∼458 eV), Ti 3p (∼38 eV), Ti 3s (∼60 eV), O 1s (∼530 eV), and C 1s (∼285 eV). The Ti 2p high-resolution spectrum (Figure 4A1) exhibited a doublet with Ti 2p_3_/_2_ and Ti 2p_1_/_2_ components centered at approximately 458.7 and 464.3 eV, respectively. These binding energies are characteristic of Ti^4+^ species, consistent with the formation of a native TiO_2_ layer on the titanium surface under ambient conditions.^35^ The O 1s spectrum (Figure 4B1) displayed a dominant peak at 530.8 eV, attributed to lattice oxygen (Ti–O) within the oxide structure. A secondary contribution at higher binding energy (532.7 and 534.3 eV) was also observed, which can be associated with surface hydroxyl groups (−OH) and/or adsorbed oxygen species. The C 1s spectrum (Figure 4C1) revealed a main peak centered at 285.5 eV, corresponding to C–C/C–H bonds from adventitious carbon contamination.^39^

**Figure 4:**
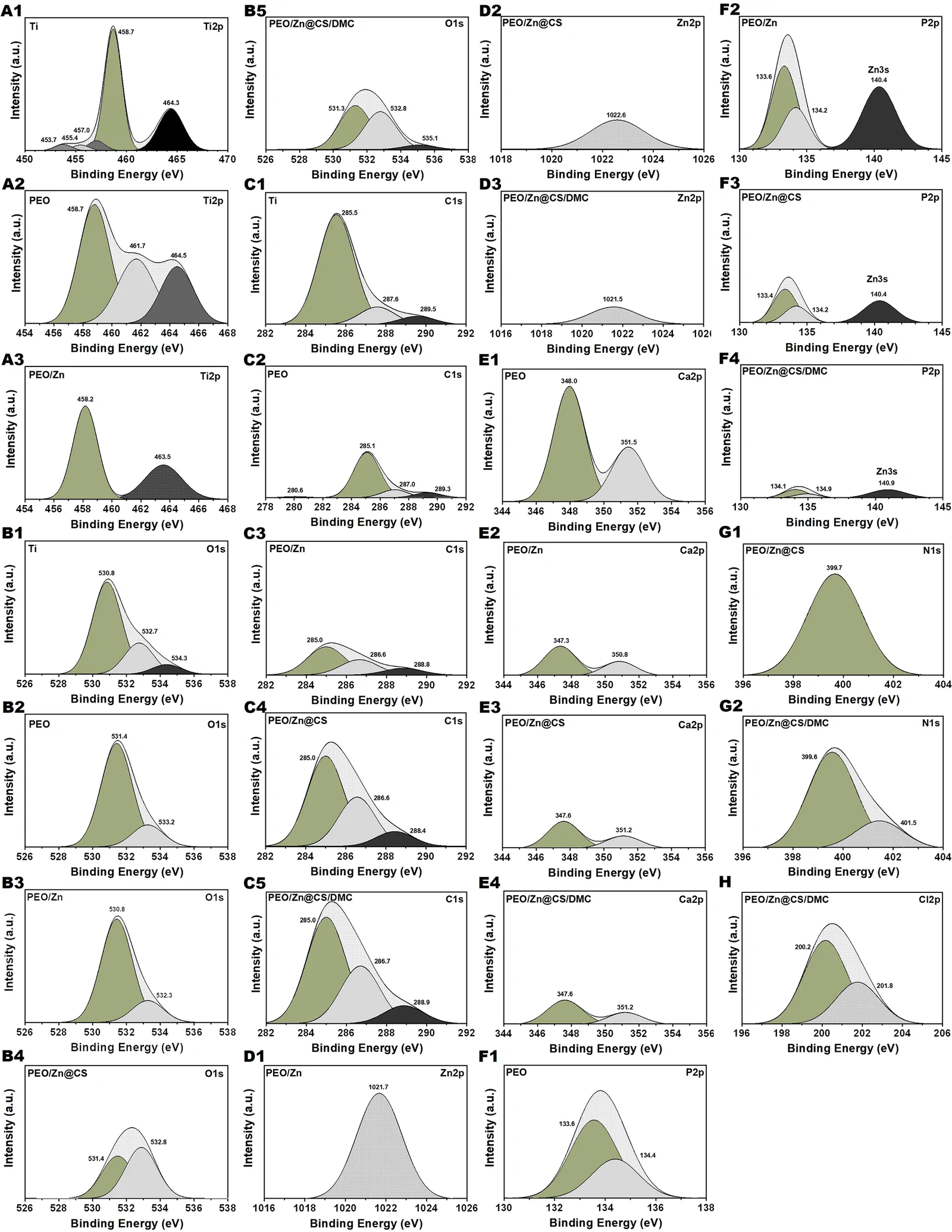
Deconvoluted high-resolution spectra showing the chemical states of the main elements detected after each surface modification step. Ti 2p spectra are shown in (A1–A3). O 1s spectra are shown in (B1–B5). C 1s spectra are shown in (C1–C5). Zn 2p spectra are shown in (D1–D3). Ca 2p spectra are shown in (E1–E4). P 2p spectra are shown in (F1–F4). N 1s spectra are shown in (G1 and G2). The Cl 2p spectrum is shown in (H).

PEO markedly altered the surface chemical profile of Ti, as evidenced by the high-resolution XPS spectra (Figure 3C). In the Ti 2p region (Figure 4A2), the Ti 2p_3_/_2_/Ti 2p_1_/_2_ doublet at 458.7/464.5 eV remained consistent with Ti^4+^ in TiO_2_, with an additional contribution at 461.7 eV likely reflecting the more complex oxide environment generated by PEO. The O 1s spectrum (Figure 4B2) showed components at 531.4 and 533.2 eV, assigned to metal–oxygen bonds within the oxide matrix and to hydroxylated or adsorbed oxygen-containing species, respectively. The C 1s region (Figure 4C2) displayed peaks at 285.1, 287.9, and 289.3 eV, corresponding to C–C/C–H, C–O/C–N, and O–C=O environments. More importantly, PEO promoted the incorporation of electrolyte-derived calcium and phosphorus, as confirmed by Ca 2p signals (Figure 4E1) at 348.0/351.5 eV and P 2p components (Figure 4F1) at 133.6/134.4 eV, indicating Ca^2+^- and phosphate-containing species in a calcium phosphate-like chemical environment.^34^

The incorporation of Zn during the PEO process further modified the surface chemical environment while preserving the general spectral features observed for the PEO group (Figure 3C). The Ti 2p region (Figure 4A3) retained components consistent with oxidized Ti species, although with attenuated Ti signals, suggesting increased surface coverage. Compared with PEO, the O 1s components (Figure 4B3) shifted to 530.8 and 532.3 eV, supporting the contribution of Zn–O bonds and/or zinc-associated hydroxylated environments within the oxide layer. Ca 2p and P 2p signals (Figure 4E2,F2) remained compatible with Ca^2+^- and phosphate-containing species, although the reduced Ca intensity indicated a lower relative surface calcium concentration after Zn incorporation. The presence of zinc was confirmed by a Zn 2p signal (Figure 4 D1) at 1021.7 eV, characteristic of Zn^2+^, together with Zn 3s at 140.4 eV (Figure 4 F3) and Zn LMM Auger features near 500 eV in the survey spectrum. Overall, these binding energies and spectral features support the incorporation of zinc into the PEO coating, most likely as ZnO within the porous oxide matrix.^34^

The deposition of a CS-based polymeric layer in the PEO/Zn@CS group further modified the surface chemistry, as evidenced by a marked attenuation of metallic and metal-oxide signals, particularly Ti 2p, Ca 2p, Zn 2p, and P 2p, thereby confirming the formation of an overlayer that shielded the underlying ceramic structure (Figure 3C). The emergence of a N 1s peak at 399.7 eV confirmed the introduction of nitrogen-containing moieties, attributable to the amide (−NH) and amine (−NH_2_) functional groups in CS. The structural integrity of the CS macromolecule was evident in the XPS data (Figure 4G1) since the presence of C–O and C–N bonds suggests retention of CS’s polysaccharide backbone of the glucosamine and N-acetylglucosamine subunits.^49^

For the PEO/Zn@CS/DMC group, the high-resolution XPS spectra (Figure 3C) exhibited nearly identical features to those of the PEO/Zn@CS group. Yet, high-resolution deconvolution of the N 1s (Figure 4G2) peak revealed contributions at 399.6 and 401.5 eV, probably corresponding to amine (−NH_2_) and protonated (−NH_3_^+^) species, respectively. This observation aligns with the C 1s and O 1s spectra, where distinct peaks associated with C–C (285 eV), C–O/C-N (286.7 eV), and C=O (288.9 eV) were identified (Figure 4C5). High-resolution deconvolution of the Cl 2p region (Figure 4H) revealed two distinct spin-orbit split components, located at 200.2 eV (Cl 2p_3_/_2_) and 201.8 eV (Cl 2p_1_/_2_). The Cl 2p_3_/_2_ transition represents the lower energy state, while Cl 2p_1_/_2_ corresponds to the higher energy component. The binding energy values are consistent with organic chloride species, particularly those found in chlortetracycline derivatives.^50^ The detection of chlorine provides direct evidence of the successful incorporation of the antibiotic within the polymeric matrix as chlorine is an exclusive component of the hydrochloride salt form of demeclocycline.

Regarding coating’s crystalline phase composition, X-ray diffraction (XRD) analysis (Figure 3D) revealed notable differences promoted by the PEO treatment. In the Ti group, only α-Ti diffraction peaks were observed, consistent with a predominantly amorphous structure.^31^ However, after the PEO treatment, the emergence of anatase and rutile phases was evident, indicating that the high-energy plasma oxidation process led to the crystallization of TiO_2_.^36^ Zn-doped PEO groups showed higher rutile intensity (Figure 3D, 2θ = 27, 36, 41°), likely due to the increased electrolyte conductivity promoted by Zn species, which stabilizes the gaseous envelope and increases localized thermal energy at the substrate–electrolyte interface.^36^ These conditions favor TiO_2_ crystallization and the anatase-to-rutile transformation, a temperature-driven process involving oxygen rearrangement and lattice structure densification. The surface crystalline phase composition is relevant for implant applications. While anatase may enhance surface bioactivity, rutile is denser and mechanically more stable due to its edge-sharing TiO_6_ octahedral arrangement.^51^ Thus, the increased rutile content and crystallinity of Zn-doped coatings may contribute to improved mechanical and wear resistance under dental and orthopedic loading conditions.^52^

Ultraviolet-Visible spectroscopy was employed to characterize the absorption profile of DMC over the spectral range corresponding to the emission wavelength of the LED system used for photodynamic activation. To replicate the conditions in which DMC is incorporated into the polymeric film, a solution with the same concentration was prepared and analyzed. The absorption spectrum (Figure 3E) exhibited a peak of approximately 0.25 au at 450 nm (Soret band), coinciding with the dominant emission wavelength of the blue-light source, confirming that DMC absorbs photons in this region. DMC’s tetracyclic structure (A, B, C, and D rings) consists of alternating single and double bonds, forming a conjugated π- electron system.^20^ This conjugation can arise from the overlap of π orbitals, allowing electrons to delocalize across the molecular framework and absorb light at specific wavelengths.^10^ When incident light matches the electronic configuration of the molecule, π-electrons in bonding molecular orbitals absorb photon energy and transition to higher-energy antibonding π orbitals. This process, known as a π-π transition, facilitates photoinduced reactions,^53^ which might be central to the photodynamic antimicrobial mechanism of DMC.

Regarding photodynamic potential, only PEO/Zn@CS/DMC exhibited significant light-mediated dye degradation, whereas the control groups and PEO/Zn@CS showed no relevant activity (Figure 3F). The response was time-dependent, reaching the highest degradation after 240 min of irradiation (18.95%). However, because prolonged irradiation is clinically impractical and may increase the risk of tissue damage, the 30 min time point was considered more relevant. At this interval, PEO/Zn@CS/DMC still produced significant dye degradation (8.17%; *p* < 0.0001) compared with all other groups (Figure 3F’), confirming the photodynamic activity of the coating. The slight degradation observed for PEO/Zn@CS, together with its high variability, may reflect dye adsorption by CS rather than true photodynamic activity.^54^ Under dark conditions, no group showed significant dye degradation (Figure 3G,G’), indicating that the effect was light-dependent and not caused by spontaneous chemical reactions.^35^ The photodynamic response of PEO/Zn@CS/DMC may be attributed to ROS generation after light activation of DMC.^17^ It is hypothesized that light-excited DMC may undergo triplet state excitation, enabling ROS generation through Type I and Type II photochemical pathways. It is plausible that the triplet-state of DMC photosensitizer can interact with surrounding molecular oxygen, initiating oxidative reactions responsible for dye degradation.^15,17^

To further verify whether the light-dependent activity of the coating was associated with ROS generation, a DCFH-DA fluorescence assay was performed (Figure 3H). Under dark conditions, all groups exhibited minimal fluorescence, indicating negligible oxidative activity in the absence of irradiation (*p* > 0.05). Upon light exposure, PEO/Zn@CS showed fluorescence levels comparable to the negative control (wells with DCFH probe without disks, maintained in the dark) whereas PEO/Zn@CS/DMC exhibited a pronounced increase in fluorescence intensity (*p* < 0.0001). Free DMC showed the highest fluorescence intensity (∼1.5 × 10^6^ au), which was also significantly higher than that of PEO/Zn@CS/DMC (*p* < 0.0001). These findings demonstrate that DMC retained its photoresponsive ROS-generating capability after incorporation into the CS-based coating, although the magnitude of the response was lower than that observed for free DMC, possibly because incorporation within the polymeric matrix partially restricts molecular mobility and interaction with the surrounding medium.

Importantly, because DCFH detects overall oxidative activity rather than individual reactive species,^55^ these results support ROS generation by PEO/Zn@CS/DMC under irradiation but do not establish the contribution of specific Type I or Type II photochemical pathways. However, previous photochemical studies support the ability of DMC to participate in both energy- and electron-transfer reactions.^15^ Singlet oxygen generation by tetracyclines has been directly proven, with DMC exhibiting particularly pronounced photosensitizing activity among tetracycline derivatives, with singlet oxygen quantum yields of 0.08.^56^ More recent studies further demonstrated that photoactivated DMC can activate probes associated with both singlet oxygen and hydroxyl radicals and can promote measurable H_2_O_2_ formation, supporting the coexistence of Type I and Type II photochemical processes.^17^ These previous observations provide a mechanistic basis for the increased DCF fluorescence detected in the present study after blue-light irradiation.

### Coating’s Mechanical Resistance

The tribological evaluation provided critical insights into the wear resistance, frictional behavior, mass loss, and drug release of the developed coatings. The macro morphology and extent of the wear scars varied significantly among the groups (Figure 5A,B). The Ti group exhibited a well-defined, concave wear track with a characteristic indentation, indicating significant penetration of the zirconia counterbody into the titanium surface (Figure 5A), leading to pronounced deformation with a total wear area measuring 5.06 mm^2^ (Figure 5B). In contrast, PEO and PEO/Zn showed the smallest wear scars, with areas of 3.12 and 2.98 mm^2^, respectively, and mainly localized maceration rather than material removal (Figure 5B). The lower wear observed under the tested conditions is consistent with the presence of a mechanically resistant ceramic-like layer that reduced damage to the underlying substrate.^31,34^ Zn incorporation further enhanced this behavior, likely by increasing coating crystallinity, consistent with the higher rutile content observed by XRD (Figure 3D).

**Figure 5:**
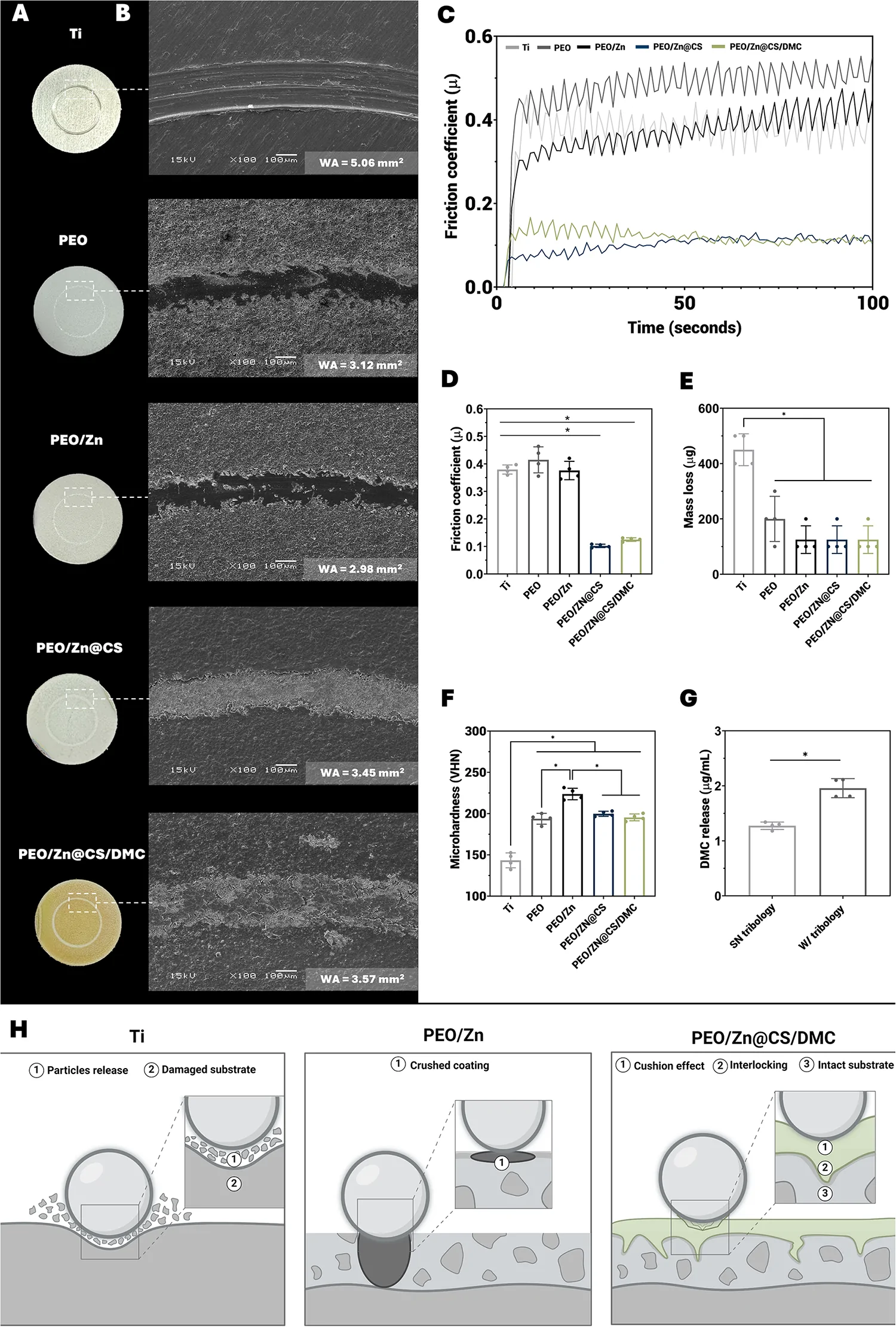
Tribological performance and mechanical properties of control and experimental coatings. (A) Macroscopic images of Ti, PEO, PEO/Zn, PEO/Zn@CS, and PEO/Zn@CS/DMC discs post-tribological. (B) SEM micrographs depicting detailed wear track morphology for each group (15 kV, scale bar = 100 μm), with quantified wear area measurements annotated. (C) Friction coefficient (μ) profiles recorded over a 100-second sliding duration. (D) Quantitative analysis of average friction coefficients (μ). (E) Total mass loss (μg) of each sample post-tribological testing. (F) Vickers microhardness values of the coatings. (G) DMC release (μg/mL) from PEO/Zn@CS/DMC coatings under tribological stress versus static. (H) Schematic illustration of tribological behavior of Ti, PEO/Zn and PEO/Zn@CS/DMC groups. Data are expressed as mean ± standard deviation. Statistically significant differences between groups are indicated by symbols, with * representing p < 0.05, as determined by Student *t* test or one-way ANOVA with Tukey’s post hoc test for multiple comparisons. Illustration created in BioRender (License number: NJ27W676G8).

In contrast, PEO/Zn@CS showed a slightly larger wear area (3.45 mm^2^) with partial delamination of the polymeric film mainly along the lateral edges of the wear track (Figure 5B). Despite this localized removal, the underlying PEO/Zn layer remained largely intact, suggesting that the CS film served as a sacrificial barrier, absorbing and dissipating tribological stress, while preserving the inner ceramic coating. PEO/Zn@CS/DMC exhibited a fracture pattern in which the polymeric coating fragmented into smaller plates rather than undergoing complete delamination.^57^ This behavior may be a result of DMC incorporation in the polymer network, increasing coating density. Importantly, the partial retention of the fragmented coating may help preserve the incorporated drug while maintaining the protection of the underlying PEO/Zn layer.

The friction coefficient analysis further clarified the role of the polymeric layer in wear mitigation (Figure 5C,D). Ti, PEO, and PEO/Zn exhibited average friction coefficients of ∼0.4–0.5, indicating stronger surface/counter-body interaction during sliding. In contrast, PEO/Zn@CS and PEO/Zn@CS/DMC showed ultra-low values of ∼0.1. This decrease, together with the lower friction amplitude, indicates that the CS-based film acted as a lubricating barrier, absorbing tribological loads and preventing direct wear of the underlying ceramic layer (Figure 5H).^30^ The mechanical stability of the inner PEO/Zn coating is also supported by its higher rutile content, whose dense crystalline structure is associated with enhanced mechanical resistance.^34^ This result correlates with the mass loss results (Figure 5E), where all PEO-treated groups exhibited significantly lower weight loss than the uncoated Ti surface (*p* < 0.05). Vickers microhardness results (Figure 5F) showed that the Ti group exhibited the lowest hardness values (∼150 VHN).^58^ The application of PEO treatment significantly increased microhardness values (*p* < 0.05), with the PEO and PEO/Zn groups reaching approximately 190 VHN and 220 VHN, respectively. Conversely, polymer-coated groups showed reduced hardness (*p* < 0.05), likely due to indentation-induced deformation of the softer polymeric layer, which may increase diagonal length and lower calculated hardness values.

The DMC release profile under tribological stress revealed that mechanical loading increased drug elution from PEO/Zn@CS/DMC (Figure 5G). The coating released 1.96 μg/mL of DMC after wear testing, significantly higher than the 1.27 μg/mL released without mechanical stress (*p* = 0.0003). This indicates that tribological forces can modulate drug release, likely through polymer disruption. However, the localized stress applied during tribological testing represents an intensified mechanical challenge, whereas in vivo forces are expected to be more broadly distributed across the implant–bone interface. Clinically, stress-responsive DMC release may be advantageous, as localized antibiotic availability during implant loading could help prevent early implant-associated infections.^59^ At the same time, partial retention of DMC within the coating is essential to preserve the photoresponsive function, ensuring that molecules remain available for antimicrobial photodynamic activity.

### Film Degradation and DMC Release

To further assess the functional performance of the coatings, the degradation behavior of the CS film and the DMC release profile were evaluated over 21 days (Figure 6). SEM analysis showed that, after 1 day of immersion in PBS, the polymeric layer remained largely preserved. After 7 days, early signs of degradation became evident, particularly in lysozyme-containing media, with gradual exposure of the PEO pores (Figure 6A). This effect became more pronounced at 21 days, when larger porous and residual degradation products were observed, especially in samples in contact with lysozyme (Figure 6A). These findings indicate progressive enzymatic degradation of CS, which is consistent with lysozyme action mediating the cleavage of β-1,4-glycosidic bonds in partially acetylated CS chains.^22^ Accordingly, the use of medium-molecular-weight CS with a degree of deacetylation of 75–85% provided a suitable balance between stability and degradability, allowing the coating to remain partially present on the surface for up to 21 days while undergoing progressive degradation and sustained DMC release during the early post-implantation period.^23^

**Figure 6:**
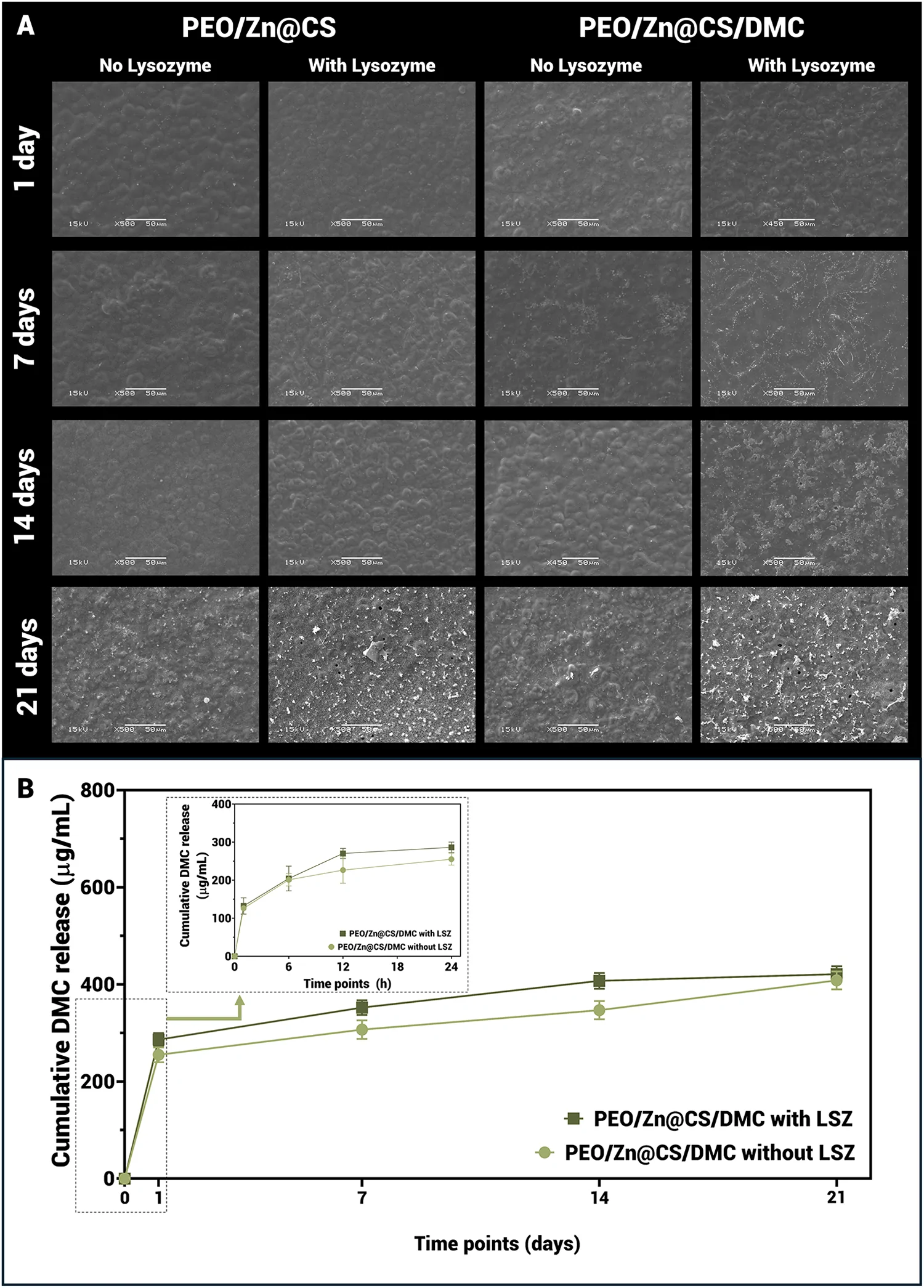
Degradation behavior of chitosan coating and demeclocycline release profile. (A) SEM micrographs (15 kV, magnification 500×, scale bar = 50 μm) depicting the surface morphology of PEO/Zn@CS and PEO/Zn@CS/DMC coatings after immersion in PBS with or without lysozyme for 1, 7, 14, and 21 days. (B) Cumulative release profile of demeclocycline from PEO/Zn@CS/DMC coatings over 1, 6, 12, and 18 h, and 1, 7, 14, and 21 days of immersion in PBS, comparing conditions with and without lysozyme. Data are presented as mean ± standard deviation.

DMC release from the CS-based film followed a biphasic pattern, characterized by an initial burst followed by sustained release. Within the first hour, approximately 100 μg/mL of DMC was released, and by 24 h, nearly half of the incorporated drug had diffused into the medium, reaching 255 μg/mL without lysozyme and 286 μg/mL with lysozyme (Figure 6B). This early release was mainly attributed to the diffusion of DMC through the hydrated CS matrix.^23,26^ In diffusion-controlled release systems, this mass-transfer process is governed by the solubilization of the drug and its subsequent movement through the polymeric network.^60^ As the polymer absorbs fluid, it undergoes a transition characterized by an expansion in volume and the formation of microvoids within the matrix, promoting an increase in the permeability of the matrix.^61^ Thereafter, cumulative release increased gradually over 21 days, reaching 408 μg/mL in PBS and 421 μg/mL under lysozyme exposure, corresponding to approximately 81.6 and 84.2% of the initially loaded DMC, respectively (Figure 6B). Overall, the release profile reflects a combined diffusion- and degradation-controlled mechanism, in which CS hydration, swelling, matrix relaxation, and enzymatic breakdown progressively increase drug release.^60,61^ The DMC availability is particularly relevant for peri-implant applications, as it may provide local antimicrobial support during the early stages of osseointegration, while residual drug retention within the coating may preserve photoresponsive activity for subsequent light-triggered antimicrobial therapy.

### Antimicrobial Effect

Peri-implant infections remain a major cause of implant failure, as bacterial colonization can impair osseointegration and compromise long-term clinical success.^1,9^ Therefore, the antimicrobial performance of DMC was first assessed through minimum inhibitory concentration (MIC)/ minimum bactericidal concentration (MBC) assays to determine the optimal DMC concentration to be incorporated into the surface. The MIC assay identified the lowest DMC concentration capable of inhibiting bacterial growth, whereas the MBC assay evaluated bacterial regrowth on antibiotic-free agar, distinguishing bacteriostatic from bactericidal activity (Figure 7A). At 4.88 μg/mL, bacterial proliferation exceeded the initial inoculum, indicating a subinhibitory concentration. From 9.77 to 312.5 μg/mL, colony-forming units (CFU) levels remained comparable to the initial inoculum, demonstrating growth arrest and confirming the bacteriostatic behavior expected for tetracycline-class antibiotics such as DMC. At 625 μg/mL, no colonies were detected after 48 h on Columbia Blood Agar, indicating the absence of viable bacteria and defining this concentration as bactericidal. Since 312.5 μg/mL already produced substantial growth inhibition, 500 μg/mL was selected for incorporation into the CS-based film to ensure effective antimicrobial loading while remaining below the bactericidal threshold (Figure 7A).

**Figure 7:**
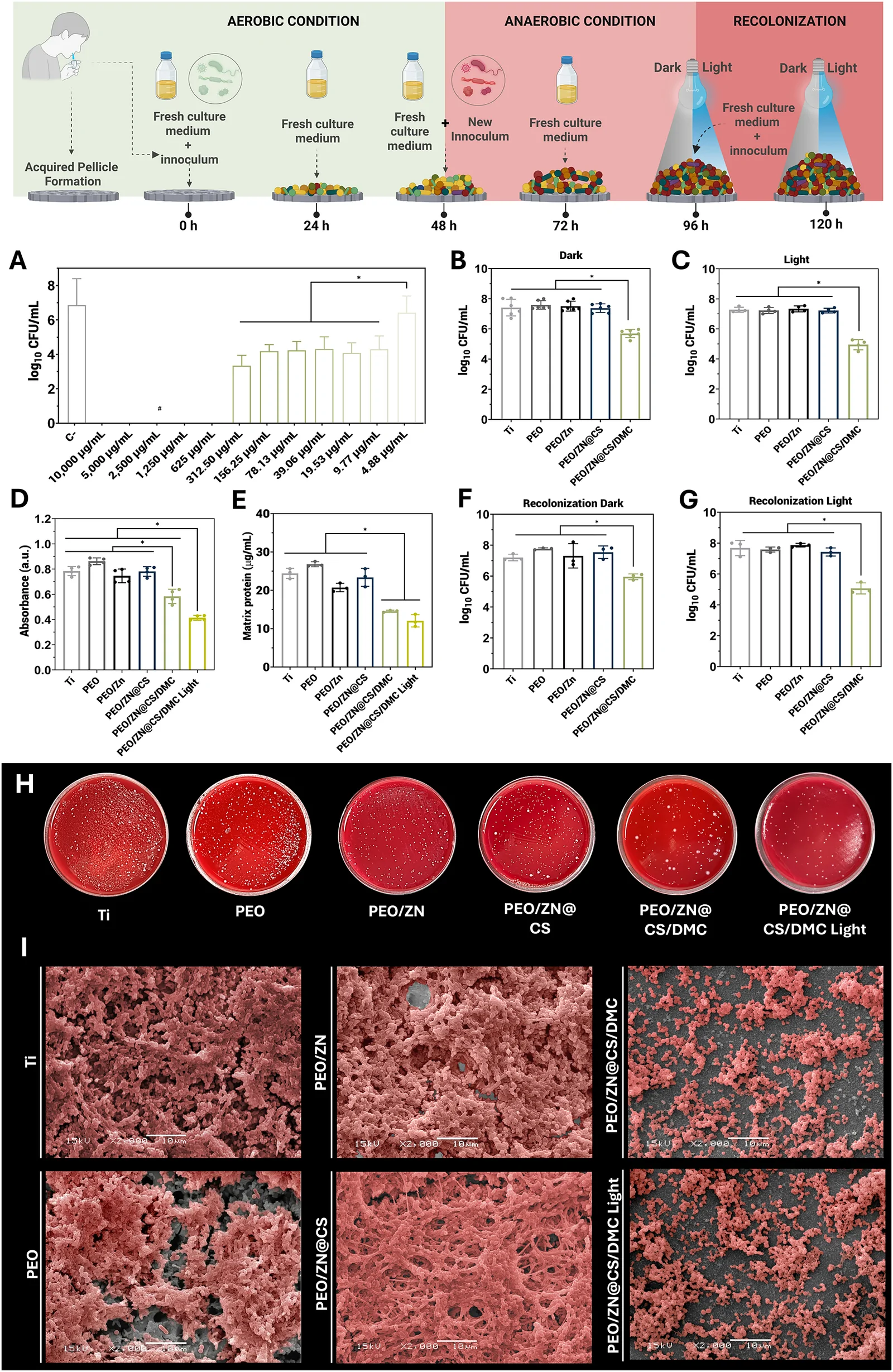
Antimicrobial methodology and performance of coatings against polymicrobial biofilms. (A) Quantification of bacterial viability for MIC and MBC determination of DMC. Bacterial viability (log_10_ CFU/mL) under dark (B) and light (C) conditions after 96 h of biofilm formation. (D) Microbial metabolic activity assessed via XTT assay. (E) Quantification of extracellular matrix protein content (μg/mL). Bacterial viability (log_10_ CFU/mL) under dark (F) and light (G) conditions following recolonization after initial biofilm eradication. (H) Representative images of CBA plates after spreading/plating of biofilm suspensions. (I) SEM micrographs (15 kV, magnification 2000×, scale bar = 10 μm) illustrating biofilm morphology. Data are expressed as mean ± standard deviation. Statistically significant differences between groups are indicated by symbols, with * representing *p* < 0.05, as determined by one-way ANOVA with Tukey’s post hoc test for multiple comparisons or one-way ANOVA with Welch correction with Games-Howell post hoc test.

The antimicrobial performance of the coatings was then evaluated using a human saliva-derived microcosm biofilm model to better reproduce polymicrobial conditions relevant to the oral environment.^62^ Although the inoculum was obtained from healthy donors, the saliva-derived microcosm protocol used here has previously been validated against clinical peri-implantitis biofilms, showing comparable microbial load, biomass, morphology, antimicrobial tolerance, and pathogenic-complex composition.^62^ Assays were performed under dark and illuminated conditions to distinguish the intrinsic antibacterial effect of DMC from its potential enhancement by photodynamic activation. Under dark conditions (Figure 7B), Ti, PEO, PEO/Zn, and PEO/Zn@CS maintained high bacterial viability, with final counts of approximately 7.5 log CFU, indicating no measurable antimicrobial effect. Although Ti and CS-coated surfaces exhibited smoother topographies, this property alone did not reduce mature biofilm formation, suggesting that surface roughness may be more influential during initial adhesion than during later biofilm maturation. Moreover, despite the reported antimicrobial properties of Zn and CS, their effects are highly concentration-dependent.^24,63^ In this four-day polymicrobial model, characterized by microbial diversity, prolonged growth, and robust biofilm architecture that may exceed 100 μm in thickness,^62^ Zn and CS, at most, acted as early adhesion modulators rather than potent microbicidal agents.

The effectiveness of PEO/Zn@CS/DMC against these challenging biofilm conditions is particularly relevant, as this group showed a significant reduction of approximately 2 log CFU/mL under dark conditions compared with the other surfaces (*p* < 0.0001; Figure 7B,C), with bacterial counts around 5.65 log CFU/mL. This effect indicates that the release of DMC from the CS matrix impaired microbial proliferation, supporting the intrinsic antibacterial function of the coating. Under illuminated conditions, Ti, PEO, PEO/Zn, and PEO/Zn@CS still exhibited extensive bacterial growth, confirming that blue light alone did not reduce the microbial viability. In contrast, blue-light irradiation of PEO/Zn@CS/DMC produced an additional reduction of approximately 1 log compared with its dark counterpart, resulting in an overall 3-log decrease in bacterial viability (*p* < 0.0001; Figure 7C). This enhanced effect is consistent with a DMC-mediated photodynamic antimicrobial mechanism, potentially involving ROS generation after photoactivation.^17^ The primary cellular targets of ROS include the lipid bilayer, intracellular proteins, and nucleic acids. In bacterial membranes, ROS initiates lipid peroxidation, leading to unsaturated fatty acid disruption and lipid hydroperoxide formation. This can compromise membrane barrier integrity, increasing its permeability, which may ultimately result in cell lysis.^11,64^ Intracellularly, ROS oxidize amino acid residues, such as cysteine, methionine, tyrosine, and histidine, impairing enzymatic activity and protein stability. They can also damage DNA through guanine oxidation and deoxyribose backbone alterations, resulting in strand breaks and impaired replication.^11,64^

The use of antibiotics as photosensitizers for aPDT may raise concerns about the potential induction of antibiotic resistance. While microorganisms display enzymatic defense mechanisms, including superoxide dismutase, catalase, and peroxidases, these enzymes are largely ineffective against ROS generated during PDT.^65^ This inefficacy is related primarily with the non-specific oxidative damage promoted on cellular membranes, leading to compromised membrane integrity and subsequent leakage of intracellular components, which impair microbial viability before resistance mechanisms can be activated.^14^ Moreover, singlet oxygen, a highly reactive form of molecular oxygen produced during PDT, presents an additional challenge for microbial defense, as no specific enzymatic pathways have evolved to neutralize this species effectively.^10^

Given that only the PEO/Zn@CS/DMC + Light group exhibited a substantial reduction in bacterial viability under illumination conditions, additional biochemical and microscopy-based analyses focused exclusively on this experimental condition and the dark-treated groups. In terms of microbial metabolism (Figure 7D), XTT assay results revealed that the presence of DMC significantly impaired bacterial metabolic activity relative to control surfaces (*p* ≤ 0.042), with a further reduction observed under light exposure (*p* = 0.03). The further metabolic reduction observed under photodynamic activation is likely attributable to ROS-mediated oxidative damage to bacterial structures.^11^ Tetracycline antibiotics primarily act by inhibiting bacterial protein synthesis through binding to the 30S ribosomal subunit and preventing aminoacyl-tRNA docking at the A-site.^20^ This interaction involves the conserved hydrophilic surface of tetracyclines with specific regions of 16S rRNA, stabilizing the antibiotic and ribosome complex and impairing codon–anticodon alignment, thereby blocking peptide elongation. Structural features within the ABCD ring system of demeclocycline may further influence ribosomal binding affinity and pharmacological behavior, potentially contributing to activity against resistant strains.^66^ This inhibition disrupts ribosomal elongation, leading to translational arrest, accumulation of misfolded proteins, and attenuation of stress response pathways.^20^

To validate this mechanism of DMC incorporation into the coating, matrix total protein quantification was performed, revealing a significant decrease in extracellular matrix (ECM)-associated proteins, from ∼25 μg/mL in other groups to below 15 μg/mL (*p* < 0.0001) in DMC-coated samples (Figure 7E). Representative CBA plate images (Figure 7H) further illustrate this effect, showing not only a marked reduction in colony number but also less dense colony growth in the DMC-treated groups. SEM micrographs (Figure 7I) supported these findings, showing extensive biofilm formation on Ti, PEO, PEO/Zn, and PEO/Zn@CS, with a dense extracellular matrix, abundant bacterial colonization, and both coccal and bacillary morphotypes. Fungal structures, including hyphae and pseudohyphae, were also observed, reinforcing the polymicrobial complexity of peri-implant biofilms.^67^ In contrast, PEO/Zn@CS/DMC surfaces showed markedly reduced biofilm coverage and matrix accumulation, which may weaken the protective biofilm barrier and increase susceptibility to mechanical debridement, chemical disinfection, and photodynamic therapy.^68^ CLSM images (Figure 8A) further confirmed this trend, showing predominantly live cells on Ti, PEO, PEO/Zn, and PEO/Zn@CS, with live-cell percentages of 90.27, 91.30, 87.87, and 90.82%, respectively (Figure 8B). In contrast, PEO/Zn@CS/DMC showed a more heterogeneous fluorescence pattern, with live cells decreasing to 65.27%. This effect was intensified after blue-light exposure, with PEO/Zn@CS/DMC + Light showing the lowest live-cell percentage (53.40%) and the highest proportion of dead cells (46.60%; Figure 8C). These findings indicate that DMC incorporation reduced bacterial viability, while light activation further increased the proportion of membrane-compromised cells, consistent with a photodynamic-mediated effect.

**Figure 8:**
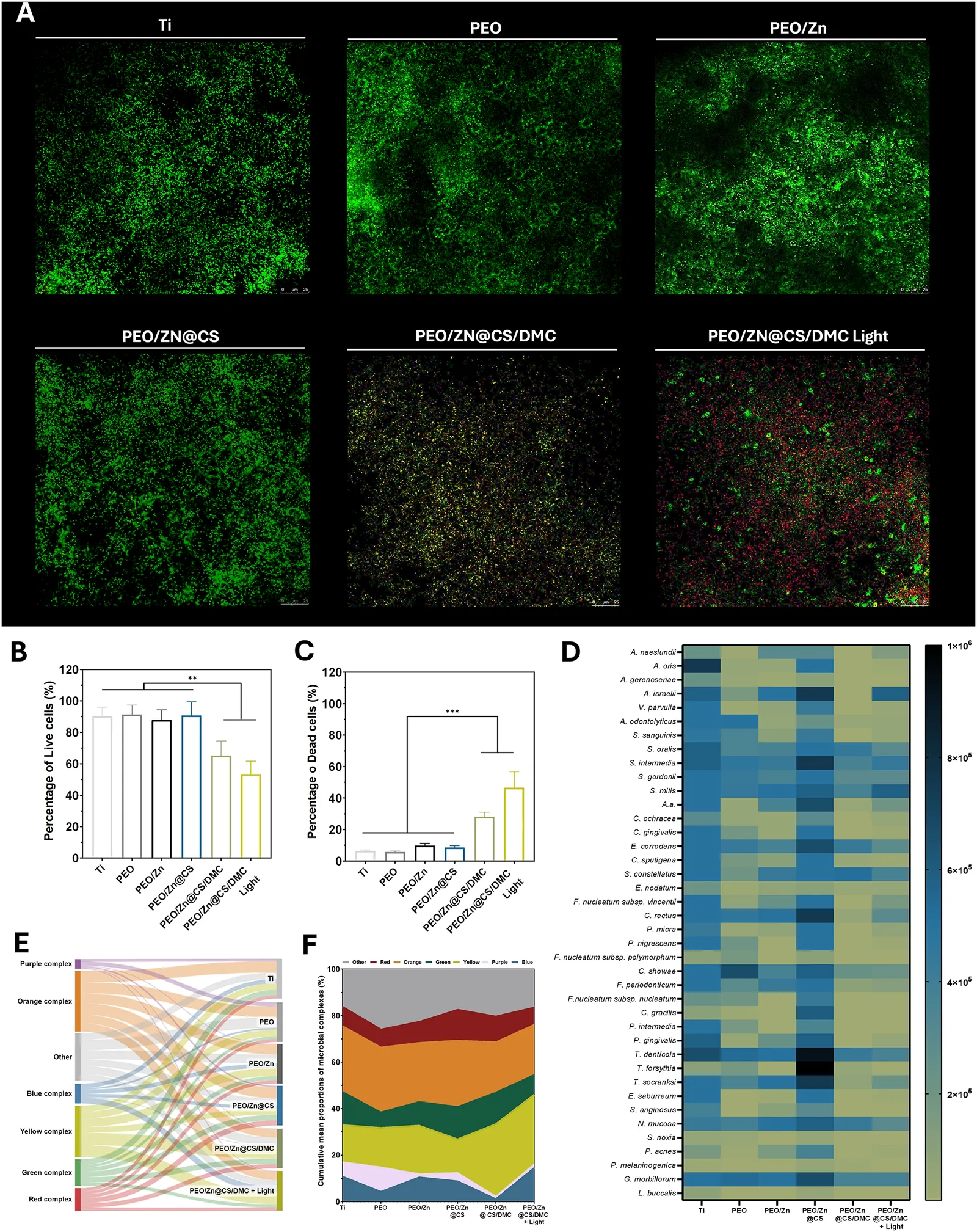
Morphology and microbial community profile of saliva-derived polymicrobial biofilms formed on modified titanium surfaces. (A) Representative confocal laser scanning microscopy (CLSM) images of Live/Dead-stained biofilms. Live cells are shown in green, whereas membrane-compromised/dead cells are shown in red. (B) Percentage of live cells and (C) percentage of dead cells quantified from CLSM images. (D) DNA–DNA checkerboard heatmap. Color intensity represents the magnitude of microbial detection, with darker shades indicating higher bacterial levels. (E) Sankey diagram; flow thickness is proportional to the relative contribution of each complex. (F) Stacked area plot showing the cumulative mean proportions of microbial complexes. For (B) and (C), data are expressed as mean ± standard deviation. Statistically significant differences between groups are indicated by symbols, with * representing *p* < 0.05, as determined by one-way ANOVA with Tukey’s post hoc test for multiple comparisons.

To assess the long-term antimicrobial functionality and resistance to recolonization, biofilms were subjected to a third inoculation cycle after 96 h. After reinoculation, Ti, PEO, PEO/Zn, and PEO/Zn@CS showed substantial biofilm regrowth, with CFU counts ranging from 7.5 to 8 log_10_ CFU/mL, indicating limited protection against renewed microbial colonization (Figure 7F). In contrast, PEO/Zn@CS/DMC maintained bacterial suppression, with counts increasing only to 5.95 log_10_ CFU/mL, which remained significantly lower than those of the control groups and confirmed the sustained antimicrobial effect of the DMC-loaded coatings. Blue-light irradiation further enhanced this response, reducing counts to 5.06 log_10_ CFU/mL in PEO/Zn@CS/DMC + light (Figure 7G), showing that the light-dependent antimicrobial effect was retained after recolonization under the tested in vitro conditions. However, the reduced magnitude of this effect compared to the initial treatment suggests gradual photosensitizer depletion from the CS matrix. Even so, the retained antimicrobial activity is relevant, as many conventional antimicrobial coatings exhibit primarily single-use efficacy and a limited capacity for repeated bacterial suppression.^37^

DNA–DNA checkerboard analysis revealed that surface modification not only modulates microbial viability but also markedly influences the composition of the polymicrobial biofilm formed on the experimental groups. The heatmap (Figure 8D) showed higher overall microbial intensities for Ti, PEO, PEO/Zn, and particularly PEO/Zn@CS, whereas the DMC groups exhibited a generalized reduction in bacterial detection, consistent with the broad-spectrum action of tetracycline antibiotics.^20^ The species-specific distribution further confirmed this pattern, since Ti, PEO, PEO/Zn, and PEO/Zn@CS supported the detection of multiple oral taxa, including early colonizers such as *Streptococcus sanguinis*, *Streptococcus oralis*, *Streptococcus mitis*, and *Actinomyces* spp., as well as anaerobic species such as *Fusobacterium nucleatum* subspecies, *Prevotella intermedia*, *Porphyromonas gingivalis*, *Tannerella forsythia*, and *Treponema denticola*. Among these groups, PEO/Zn@CS tended to show higher signals for several species, suggesting that the CS film alone did not prevent polymicrobial colonization. In contrast, DMC coatings shifted the profiles toward lower bacterial levels, with the light-activated PEO/Zn@CS/DMC group showing the most consistent reduction across the panel.

The distribution of microbial complexes showed that DMC-containing coatings altered the ecological profile of the polymicrobial biofilm (Figure 8E,F). The Sankey diagram (Figure 8E) illustrates the proportional flow of each microbial complex toward the different surfaces, with thicker bands indicating higher relative contributions. In Ti, PEO, PEO/Zn, and PEO/Zn@CS, orange-complex species represented the largest fraction, ranging from 25.4 to 28.5%, while PEO/Zn@CS showed the highest red-complex proportion (13.3%), indicating that CS alone did not prevent the enrichment of peri-implantopathogenic taxa. In contrast, PEO/Zn@CS/DMC increased the yellow-complex fraction to 31.2% and reduced the combined orange/red complexes from 41.8% in PEO/Zn@CS to 32.7%. After blue-light activation, PEO/Zn@CS/DMC + Light maintained a high yellow-complex proportion (30.0%) and further reduced orange/red complexes to 29.0%, the lowest among all groups (Figure 8F). This ecological shift suggests that DMC, particularly after photoactivation, limited dysbiotic biofilm maturation, likely through direct antibacterial activity and reduced matrix accumulation, which may restrict anaerobic niches favorable to peri-implant pathogens.

Despite these promising antimicrobial effects, the use of DMC as a conventional antibiotic may raise concerns about resistance selection. From a long-term microbiological perspective, repeated exposure to subinhibitory DMC concentrations could select less susceptible subpopulations. However, this risk may be mitigated since DMC provides broad-spectrum antibacterial activity and, upon light activation, generates ROS that damage multiple cellular targets, thereby reducing the reliance on a single antibiotic mechanism. Moreover, its localized incorporation into a degradable chitosan layer concentrates the therapeutic effect at the biofilm interface while limiting systemic exposure and prolonged drug release. Also, efflux pumps are an important mechanism of bacterial resistance to tetracyclines, because they reduce intracellular antibiotic concentrations.^69^ Nevertheless, DMC expelled from bacterial cells may remain within the biofilm microenvironment and retain its photosensitizing activity. However, this hypothesis requires direct experimental validation and does not exclude other resistance mechanisms such as ribosomal protection or enzymatic inactivation.^70^ Although localized delivery and photoactivation may mitigate this risk, they do not eliminate it.

From a translational perspective, light could be delivered in a similar way to conventional aPDT protocols for peri-implantitis.^12,13^ In nonsurgical treatment, narrow optical fibers or LED tips can be introduced into the peri-implant pocket and positioned adjacent to the exposed and contaminated implant threads.^71^ In surgical treatment, flap elevation and granulation tissue removal provide direct access to the implant surface, further facilitating controlled light delivery.^72^ The proposed coating could follow a similar irradiation principle while potentially reducing the need for repeated application of an external photosensitizer since DMC is already incorporated into the implant-associated polymeric layer. Therefore, the proposed system is intended for direct, localized activation of exposed implant surfaces during peri-implantitis treatment rather than for trans-tissue irradiation. Nevertheless, future studies should optimize clinically compatible light delivery parameters, including irradiation time, fluence, fiber geometry, and access to different implant regions.

### Bioactivity and Cytocompatibility

Beyond antimicrobial performance, implant coatings must preserve favorable surface properties that support early tissue integration, including biomineralization potential, cellular compatibility, and protein adsorption. In this sense, the ability of implant surfaces to promote hydroxyapatite (HAp) formation is commonly used as an in vitro indicator of biomineralization potential.^31^ After 28 days immersed in simulated body fluid (SBF), samples from all groups exhibited HAp deposition, although with distinct morphological patterns according to the surface treatment (Figure 9A). Ti displayed granular HAp deposits arranged in irregular clusters, with areas of exposed substrate, suggesting limited nucleation efficiency due to the absence of microstructural features or bioactive elements favoring calcium phosphate precipitation. This was supported by XRD, which showed a single diffraction peak at approximately 45°, consistent with calcium phosphate formation (Figure 9B).^31^ PEO treatment markedly enhanced mineral deposition, producing extensive plate-like HAp structures with epitaxial growth and near-complete surface coverage. This response is likely associated with the increased roughness and porous architecture generated by PEO, which provides nucleation sites for calcium phosphate crystallization.^30,34^ XRD confirmed HAp formation, showing a pattern similar to Ti. The PEO/Zn group exhibited complete surface coverage with smaller, quadrangular, plate-like HAp structures, suggesting that Zn incorporation further promoted mineral deposition. This effect may be related to changes in ion exchange and surface charge, which could favor calcium phosphate precipitation.^34,73^ Consistently, XRD revealed three HAp-related peaks at 32, 41, and 45° (Figure 9B).^31^

**Figure 9:**
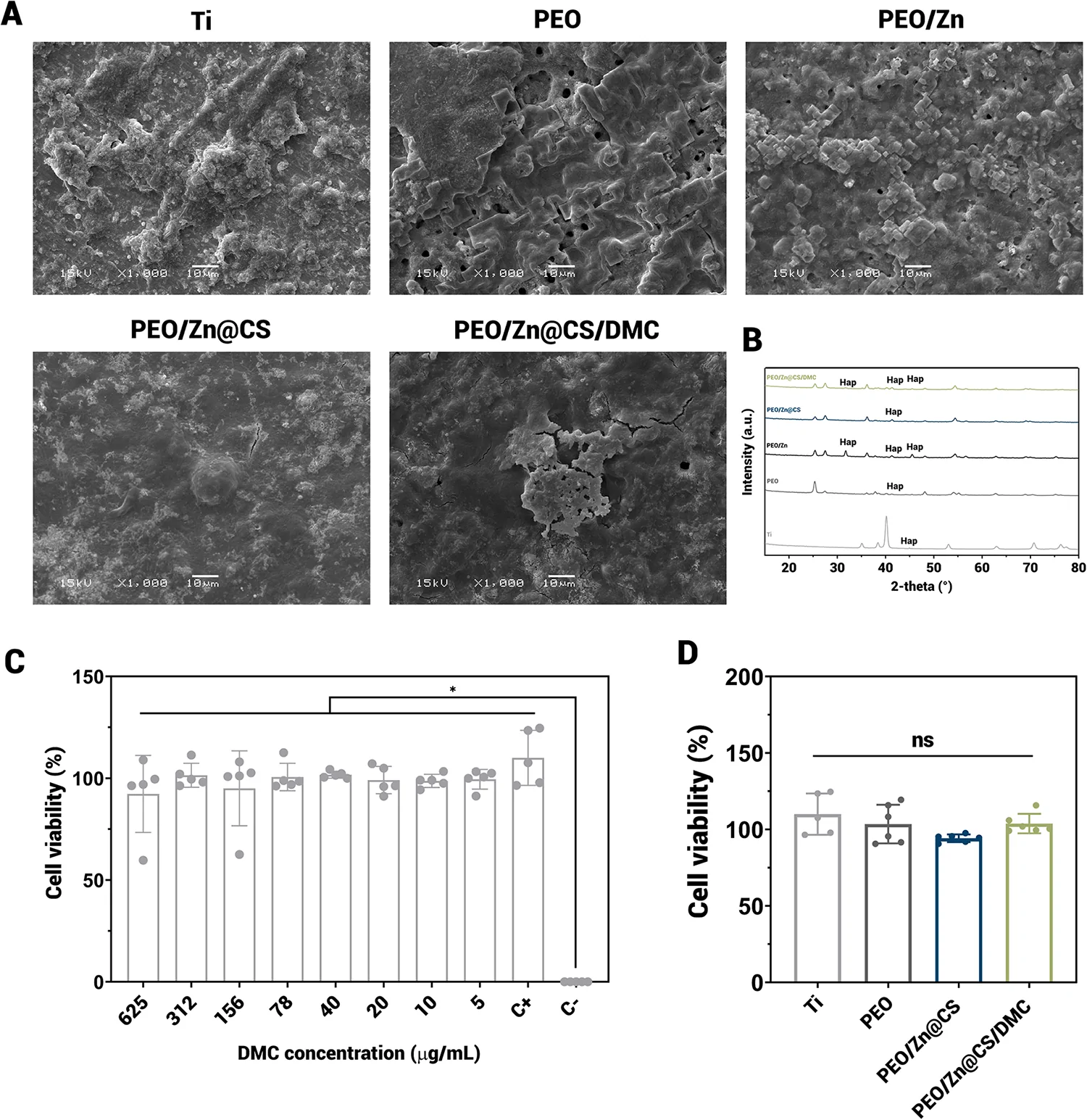
Bioactivity and cytocompatibility assessment. (A) SEM micrographs (15 kV, magnification 1000×, scale bar = 10 μm) illustrating hydroxyapatite formation on surfaces after 28 days of immersion in SBF. (B) XRD patterns indicating crystalline phases associated with hydroxyapatite deposition on the different coatings. (C) Viability of pre-osteoblastic cells after 24-hour direct exposure to various concentrations of DMC. (D) Viability of pre-osteoblastic cells cultured indirectly with extracts from Ti, PEO, PEO/Zn, PEO/Zn@CS, and PEO/Zn@CS/DMC coatings. Data are presented as mean ± standard deviation. Statistical analysis was performed using one-way ANOVA with Tukey’s post hoc test, with *p* < 0.05 considered statistically significant.

For PEO/Zn@CS, HAp deposition was thinner and more heterogeneously distributed, likely because the CS film reduced direct exposure of the underlying PEO/Zn layer and its Ca/P-containing surface to the surrounding medium. Nevertheless, the diffraction peak at 41° confirmed that biomineralization still occurred, indicating that the polymeric coating modulated but did not prevent HAp formation (Figure 9A,B).^31^ In contrast, PEO/Zn@CS/DMC showed a distinct mineralization pattern, characterized by calcium phosphate nano-flake-like structures. This response may be explained by the affinity of demeclocycline, as a tetracycline derivative, for divalent cations such as calcium.^66^ DMC may enhance local calcium retention, increasing calcium phosphate supersaturation at the interface and favoring precipitation. Together, these findings indicate that PEO/Zn@CS/DMC coatings preserved biomineralization potential while integrating antimicrobial and photoresponsive functionality.

The cellular response to implant surfaces is also critical for osseointegration, particularly regarding the viability and functionality of pre-osteoblastic cells exposed to implant coatings. Pre-osteoblastic cell viability was assessed by the AlamarBlue metabolic activity assay to determine the direct cytocompatibility of DMC and the indirect cytocompatibility of coating extracts. First, the direct cytotoxicity of DMC was evaluated by exposing pre-osteoblastic cells to DMC at concentrations selected based on previously determined MBC values. After 24 h, all tested DMC concentrations maintained cell viability above the 70% threshold defined by ISO 10993-5, indicating no acute cytotoxicity (Figure 9C). These findings are consistent with previous reports showing that tetracycline-class antibiotics, including DMC, do not induce significant membrane damage in osteoblastic cells, as indicated by the absence of LDH release even at concentrations up to 400 μg/mL.^74^ The second phase evaluated the indirect cytocompatibility of the coatings using extracts obtained from the coated disks. None of the tested surfaces, including PEO, PEO/Zn@CS, and PEO/Zn@CS/DMC, caused a statistically significant reduction in pre-osteoblastic cell viability compared with uncoated Ti (*p* ≥ 0.062; Figure 9D). This indicates that the coating extracts did not reduce pre-osteoblastic cell viability under the tested conditions.

### Proteomic Profile

Because implant surfaces are rapidly conditioned by proteins upon contact with biological fluids,^75^ we next evaluated the plasma-derived proteomic profiles formed on the experimental coatings. Mass spectrometry-based proteomic analysis showed that unprocessed plasma contained 438 identified proteins, whereas the material surfaces adsorbed a slightly lower and comparable number of proteins: 358 on Ti, 338 on PEO, 332 on PEO/Zn, 346 on PEO/Zn@CS, and 357 on PEO/Zn@CS/DMC (Figure 10A,B). The full list of identified proteins can be consulted in Table S1. Venn diagram analysis (Figure 10A) revealed 284 proteins shared across all groups, indicating a conserved adsorption core largely preserved despite surface modification. In contrast, group-specific proteins were limited, with 139 exclusive proteins in plasma, three in Ti, and 6 in PEO/Zn@CS/DMC, while no exclusive proteins were detected for PEO, PEO/Zn, or PEO/Zn@CS. The exclusive proteins identified on PEO/Zn@CS/DMC included, namely, F-actin cross-linking protein (A0A7I2 V4Y4), cofilin-1 (E9PK25), phosphoglycerate kinase 1 (P00558), tubulin β chain (P07437), heat shock protein HSP90-β (P08238), and tubulin α-1A chain (Q71U36), suggesting that DMC functionalization introduced only subtle selectivity in protein adsorption rather than a major reshaping of the plasma-derived proteome.

**Figure 10:**
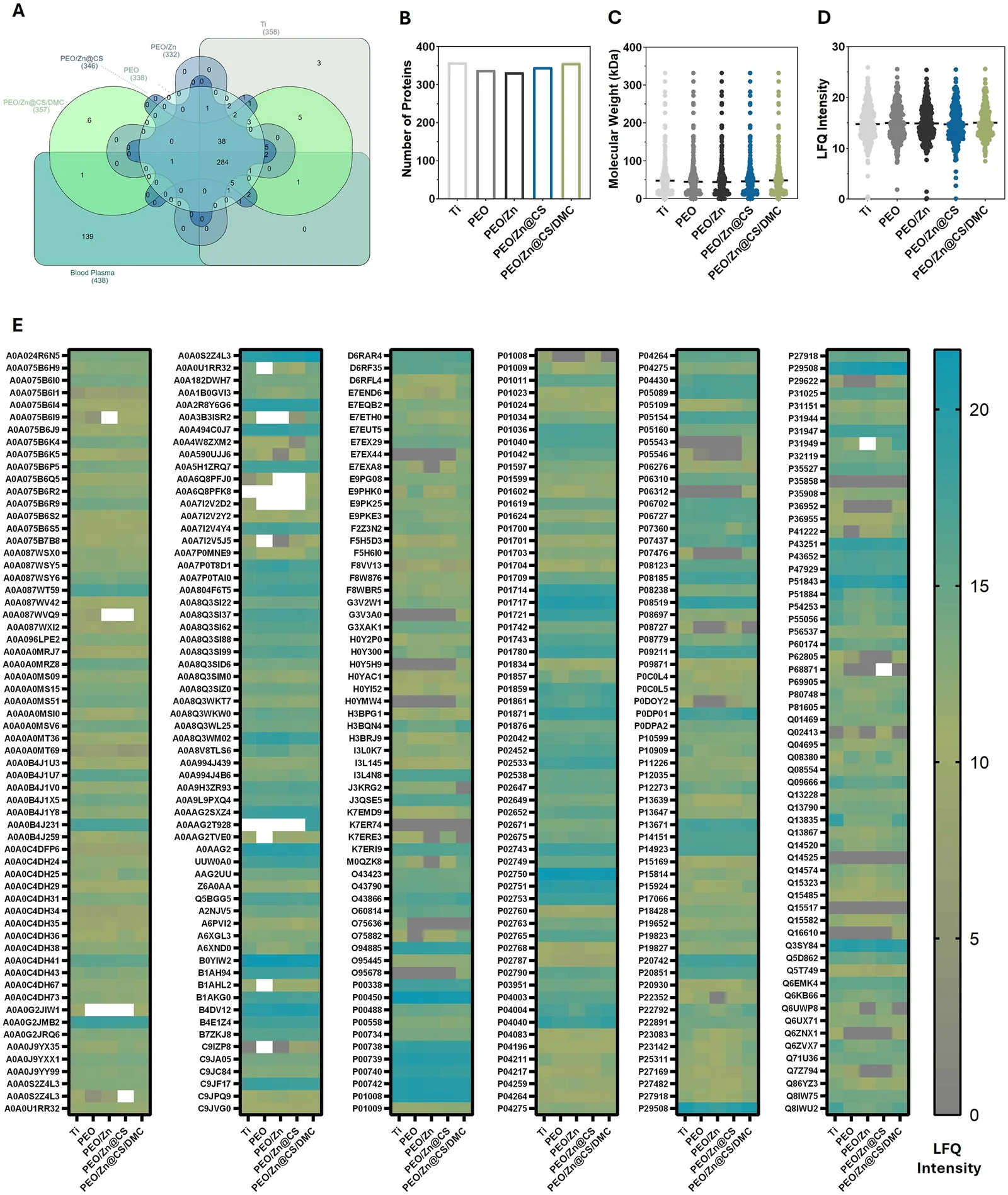
Plasma protein adsorption profile on titanium and modified surfaces. (A) Venn diagram of proteins identified from human blood plasma. (B) Number of proteins identified on each surface after plasma exposure. (C) Distribution of molecular weight values of the adsorbed proteins. (D) Distribution of label-free quantification (LFQ) intensity values, representing relative protein abundance on each surface. (E) Heatmap of LFQ intensities for the proteins detected. Color intensity represents protein abundance, with higher LFQ values indicating greater relative abundance.

The molecular weight distribution of adsorbed proteins was generally comparable among surfaces, with median values ranging from 30.81 kDa for PEO/Zn to 38.71 kDa for PEO/Zn@CS/DMC (Figure 10C). PEO/Zn showed a shift toward lower-molecular-weight proteins, whereas Ti and PEO/Zn@CS/DMC displayed slightly higher median values. LFQ intensity distributions were highly similar across groups, with median values ranging from 14.41 to 14.53, indicating comparable protein abundance profiles (Figure 10D). Consistently, the LFQ heatmap showed a predominantly conserved abundance pattern across all groups (Figure 10E). Although localized enrichment or depletion was observed for specific proteins, these differences were not widely common and did not generate clear clustering, which reinforces that plasma protein adsorption was dominated by a shared core proteome with only surface-dependent variations.

Protein–protein interaction (PPI) network analysis using STRING revealed highly interconnected proteomic profiles across all groups (Figure 11A), indicating that the identified protein sets contained significantly more known interactions than expected by chance. Groups presented similar enriched networks: Ti (245 nodes/454 edges); PEO (233 nodes/441 edges); PEO/Zn (225 nodes/440 edges); PEO/Zn@CS (237 nodes/451 edges); and PEO/Zn@CS/DMC (245 nodes/467 edges). All networks exhibited PPI enrichment *p* values < 1.0 × 10^–16^, demonstrating significantly more interactions than expected for random protein sets of comparable size. Functional enrichment analyses were then performed to determine the biological relevance of the adsorbed protein layers. Here, the comparison focused on Ti and PEO/Zn@CS/DMC, representing the baseline and fully functionalized coating, respectively. PPI networks for PEO, PEO/Zn, and PEO/Zn@CS are shown in Figures S1–S3 (Supporting Information).

**Figure 11:**
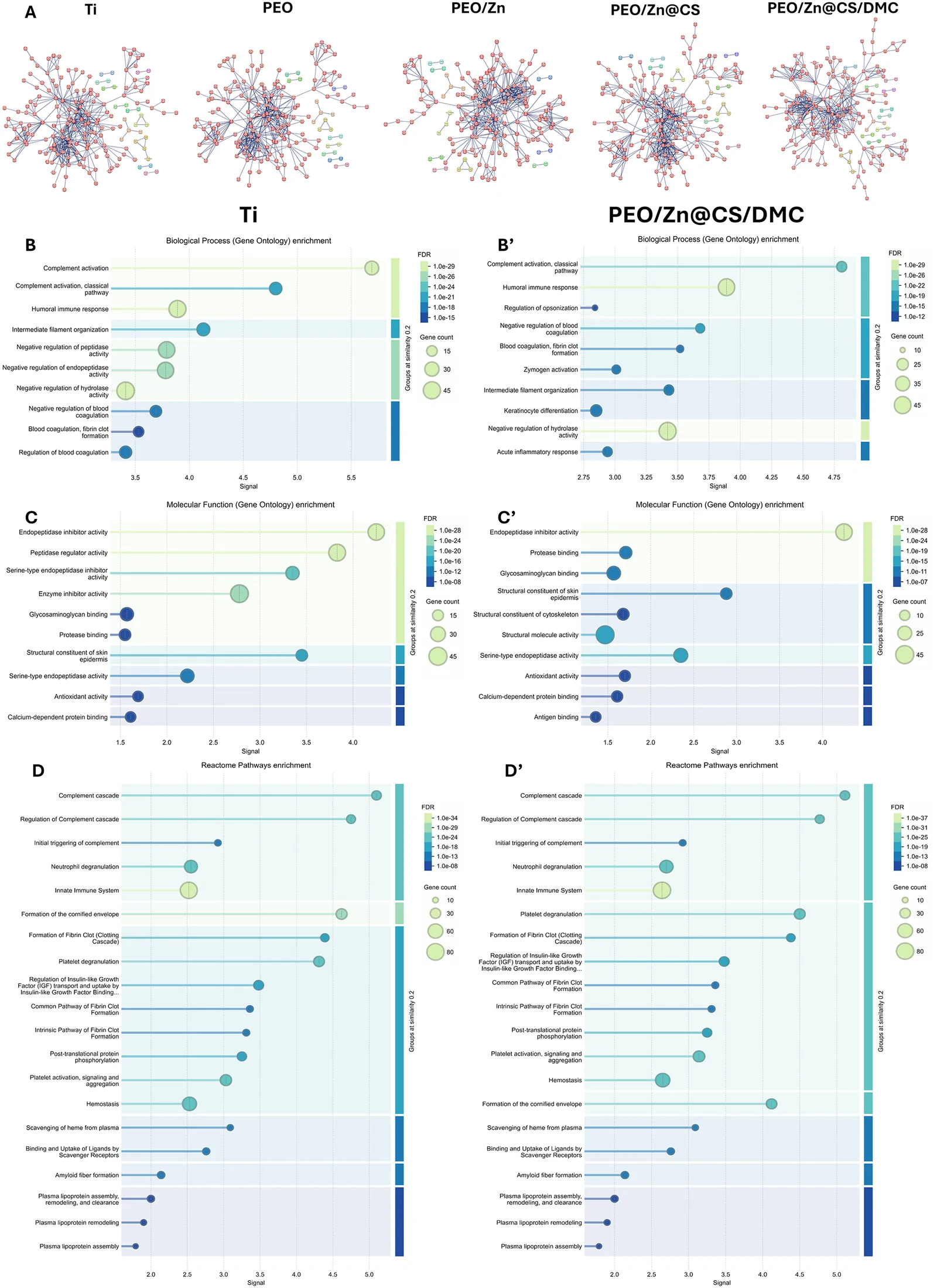
Protein–protein interaction networks and functional enrichment of plasma-derived proteins adsorbed onto surfaces. (A) Protein–protein interaction (PPI) networks generated using STRING for proteins adsorbed. (B, B’) Gene Ontology biological process enrichment. (C, C’) Gene Ontology molecular function enrichment. (D, D’) Reactome pathway enrichment. Bubble size represents gene count, color scale indicates false discovery rate (FDR), and the *x*-axis represents enrichment signal.

Subtle differences appeared in the relative contribution of specific biological processes. PEO/Zn@CS/DMC showed stronger enrichment of terms related to coagulation, including blood coagulation, fibrin clot formation, and zymogen activation, suggesting enhanced interaction with hemostatic proteins (Figure 11B,B’). Both surfaces also enriched intermediate filament organization and acute inflammatory response, indicating the adsorption of structural, cytoskeleton-associated, and immune-related proteins. However, no biological process uniquely defined either group, indicating that surface modification did not promote a fundamentally distinct biological identity but rather modulated the relative enrichment of already shared pathways. Molecular function enrichment revealed a highly overlapping profile between Ti and PEO/Zn@CS/DMC (Figure 11C,C’). Endopeptidase inhibitor activity was among the most enriched functions in both groups, indicating a strong contribution of protease-regulatory proteins to the adsorbed layer. Additional functions, including serine-type endopeptidase activity, complement binding, antioxidant activity, glycosaminoglycan binding, and calcium-dependent protein binding, were similarly enriched in both surfaces.

Reactome pathway analysis confirmed a conserved biological functionality dominated by complement activation, innate immunity, and hemostasis (Figure 11D,D’). In both groups, the complement cascade was among the most enriched pathways, accompanied by initial triggering of complement, C3/C5 activation, and broader innate immune system terms, in accordance with the central role of immune surveillance in early host–material interactions. Pathways related to hemostasis, including fibrin clot formation and coagulation cascades, were also strongly represented, reinforcing the close interplay between immune activation and coagulation at the biomaterial interface. These findings suggest that the PEO/Zn@CS/DMC surface does not fundamentally alter the biological identity of the proteomic profile, but rather modestly influences functional categories of biological responses at the implant interface.

Protein adsorption is governed by the combined effects of surface chemistry, roughness, wettability, charge, and structural organization, which determine the protein affinity, competitive exchange, and conformational rearrangement at the interface.^76^ It is believed that increasing surface roughness increases the adsorption of proteins by creating additional adsorption sites, pores, and topographical features that can physically retain proteins.^76–78^ On titanium surfaces, increased hydrophilicity may also favor protein adsorption because hydroxyl-rich surfaces exhibit higher surface free energy and provide additional sites for electrostatic interactions and hydrogen bonding. These interfacial conditions can enhance the adsorption and stabilization of proteins such as albumin and fibronectin.^76^ Also, more hydrophobic surfaces may preferentially adsorb proteins associated with complement activation and acute inflammatory pathways, potentially because stronger hydrophobic interactions promote protein binding and conformational rearrangement.^79^ Surface chemistry can also influence protein adsorption since the incorporation of divalent ions such as Ca^2+^ and Mg^2+^ into titanium surfaces or coatings can enhance protein adsorption through electrostatic bridging. These cations can coordinate simultaneously with negatively charged surface groups and anionic residues on proteins, reducing the electrostatic repulsion and stabilizing protein attachment at the interface.^76^

In the present study, these surface features likely balanced each other. PEO produced the roughest and most hydrophilic surfaces, increasing the available adsorption area and the density of hydroxyl-rich sites, whereas Zn incorporation modified the oxide chemistry and introduced additional ionic interactions. A previous study demonstrated that increasing Zn incorporation into titanium surfaces promoted greater adsorption of blood plasma proteins, supporting a concentration-dependent effect of surface chemistry.^34^ In contrast, CS deposition partially filled the porous topography, reduced the roughness, and slightly increased the contact angle, which would be expected to limit physical retention and weaken some water-mediated interactions. At the same time, however, the polymeric layer introduced amino and hydroxyl groups capable of potentially forming hydrogen bonds and electrostatic interactions with plasma proteins. Thus, the reduction in the topographical area and hydrophilicity was counterbalanced by the new chemical functionalities of CS and DMC. This balance likely explains why the total number and abundance of adsorbed proteins remained broadly similar among groups, while only subtle differences in protein selectivity and pathway enrichment were observed.

Immediately after implantation, titanium surfaces are rapidly conditioned by abundant plasma proteins that mediate the initial host response. Albumin is among the earliest adsorbed proteins because of its high plasma concentration and may contribute to the attenuation of acute inflammatory cell recruitment. In contrast, fibrinogen participates directly in coagulation and can promote platelet adhesion and activation.^76,79^ The stronger enrichment of blood coagulation and fibrin clot formation on PEO/Zn@CS/DMC indicates a greater representation of hemostasis-related proteins on the fully functionalized surface. Yet, the presence of adhesive glycoproteins such as fibronectin and vitronectin is particularly relevant to tissue integration, as their RGD-containing domains support integrin-mediated adhesion and spreading of osteogenic cells.^80^ Control and experimental surfaces also adsorb coagulation factors, including prothrombin and factors V, X, and XI, together with complement components such as C3 and C1s.^76,81^ Therefore, the adsorbed protein layer does not merely reflect passive surface conditioning but establishes the molecular interface that regulates hemostasis, immune recognition, cell adhesion, and subsequent tissue integration.

## Limitations and Future Directions

Despite the comprehensive nature of this study, limitations should be considered. The experiments were conducted under *in vitro* conditions, which cannot fully replicate the complex and dynamic *in vivo* environment. Thus, *in situ* and *in vivo* investigations are necessary to elucidate the coating’s behavior and clinical efficacy in more biologically complex scenarios. Another limitation is the temporal scope of the degradation and drug release analyses, which were limited to 21 days. Although this time point aligns with the window of early osseointegration, it limits a comprehensive understanding of the coating’s long-term performance. Prolonged evaluations are necessary to determine the full extent of DMC release, its residual antimicrobial activity over time, and potential necessary adjustments in drug concentration to optimize antimicrobial efficacy. Moreover, the coating was optimized for early-stage peri-implant infection management by using the degradation kinetics of CS to enable timely DMC release during initial healing. For prolonged antimicrobial applications, future research should explore strategies to modulate the interaction between drug and polymer, such as crosslinking agents or encapsulation techniques, to decelerate drug release and extend photodynamic efficacy. Although DMC combines broad-spectrum antibacterial activity with a multi-target photodynamic mechanism, these features do not eliminate the risk of resistance selection. Future studies should therefore assess changes in antimicrobial susceptibility and resistance-associated genes following repeated exposure and photoactivation cycles. Also, it should be acknowledged that tribological testing does not fully simulate implant insertion and long-term functional loading, which should be addressed in future studies using threaded implants and bone-mimicking substrates. Lastly, although preliminary bioactivity results are promising, further studies are essential to assess long-term cellular proliferation, differentiation, and mineralization on coated surfaces.

## Conclusions

Here, a photoresponsive bilayer coating was designed to provide localized and on-demand antimicrobial activity for the implant surfaces. The porous PEO/Zn layer provided a mechanically resistant anchoring interface for the polymeric film, while the CS matrix allowed for DMC incorporation and sustained release. Functionally, DMC release significantly inhibited polymicrobial biofilm formation by reducing bacterial viability, metabolic activity, extracellular matrix accumulation, and abundance of peri-implantopathogenic microbial complexes. Upon blue light irradiation, the coating exhibited enhanced antimicrobial efficacy. Importantly, this response was retained after biofilm recolonization, indicating repeated antimicrobial functionality and sustained resistance to microbial regrowth. Beyond infection control, the coating preserved preosteoblastic cell viability, supported hydroxyapatite nucleation, and maintained a blood plasma protein adsorption profile favorable to subsequent biointerface events. Overall, the integration of a Zn-doped porous oxide anchoring a DMC-loaded CS film represents a promising multifunctional strategy to mitigate early peri-implant infections while preserving biological features relevant to osseointegration.

## Experimental Section

This *in vitro* study aimed to evaluate the properties and bioactivity of a novel bilayer coating for dental implants, as illustrated in Figure 1. Commercially pure Ti disks were selected as substrates and divided into five groups according to their surface treatments. The first control group (Ti) comprised Ti disks that were mechanically polished. The second control group (PEO) received PEO treatment, resulting in a porous, ceramic-like surface enriched with calcium and phosphorus that served as a porous surface control. The first experimental group (PEO/Zn) underwent a similar PEO process with the incorporation of zinc ions to further enhance the resistance and bioactivity of the coating. The second experimental group (PEO/Zn@CS) combined the PEO/Zn treatment with the deposition of a CS polymeric layer designed to provide drug release. Finally, the third experimental group (PEO/Zn@CS/DMC) incorporated demeclocycline into the CS layer to enhance antimicrobial efficacy and impart photoresponsiveness to the surface. To ensure a comprehensive understanding of the structural, mechanical, and biological properties of the developed coatings, the study employed an experimental protocol encompassing advanced physicochemical characterization, mechanical and tribological assessments, microbiological investigations, proteomic analysis, and biological assays to elucidate their potential to combat infection and cytocompatibility.

### Substrate Preparation

To ensure a consistent baseline for subsequent treatments, grade II commercially pure Ti disks (Realum Industria e Comercio de Metais Puros e Ligas Ltd., Saõ Paulo, SP, Brazil) were subjected to a standardized preparation process. The surfaces of the disks were initially polished using #320- and #400-grit silicon carbide (SiC) abrasive papers (Carbimet 2, Buehler, Lake Bluff, IL) with an automatic polishing system (EcoMet/AutoMet 250 Pro, Buehler). After polishing, disks were immersed in an ultrasonic bath containing deionized water for 10 min to remove debris and contaminants. Subsequently, the samples were cleaned by immersion in 70% isopropanol for an additional 10 min, then dried before proceeding to the experimental surface treatments.^35^

### Porous Inner Layer Preparation

The inner layer was prepared using a PEO technique, which employed a pulsed direct current (DC) power supply (Plasma Technology Ltd., Kowloon, Hong Kong, China). The Ti disks acted as the anode of the system and were submerged in an electrolyte solution contained in a stainless-steel tank serving as the cathode. The system included a cooling mechanism to maintain a stable temperature of 23 ± 1.5 °C throughout the process. The electrolyte solution (1 L) was prepared in two stages. First, 0.015 M disodium ethylenediaminetetraacetic acid (Na_2_(EDTA), 99% purity; Dinâmica Ltd., Brazil) was dissolved in 500 mL of distilled water under constant agitation. To this solution, 0.1 M zinc acetate [Zn­(CH_3_COO)_2_·2H_2_O, 99% purity; Synth Ltd., Brazil] was incrementally added at room temperature. Separately, 0.1 M calcium acetate [Ca­(CH_3_COO)_2_·H_2_O, 99% purity; Dinâmica Ltd., Brazil] and 0.03 M glycerol phosphate disodium salt hydrate (C_3_H_7_Na_2_O_6_P·H_2_O, 99% purity; Sigma-Aldrich, USA) were dissolved in 500 mL of distilled water each. The two solutions were subsequently combined to create the final electrolyte, ensuring complete dissolution of all reagents and solution stability before use. The PEO process was conducted for 7 min, with the power supply parameters set to 500 V, 800 Hz, and an 8% duty cycle. These parameters and electrolyte solution composition were employed based on a previous study^34^ to achieve highly resistant coatings. Upon completion of the PEO treatment, the samples were thoroughly rinsed with deionized water to remove residual electrolyte, air-dried, and stored for subsequent modifications and analyses.

### Polymeric Outer Layer Deposition

For the deposition of the polymeric layer, disks from the PEO/Zn groups served as substrates. All experimental procedures were conducted under sterile conditions using sterilized or purified reagents to prevent contamination. Prior to polymeric layer deposition, the PEO/Zn-coated disks were sterilized by UV-light exposure (4 W, λ = 280 nm, Osram Ltd., Berlin, Germany) for 20 min on each side.^34^ The polymeric film was prepared using medium molecular weight CS (C_12_H_24_N_2_O_9_, Sigma-Aldrich, St. Louis, MO, USA), dissolved in a 0.2% (v/v) acetic acid (C_2_H_4_O_2_) solution to achieve a final concentration of 2 mg/mL.^29,40^ For the PEO/Zn@CS group, 80 μL of the prepared CS solution was carefully dispensed onto the disk surface.^82^ After applying the initial layer, the coated disks were transferred to an incubator at 37°C. Upon complete drying, the deposition process was repeated to form a second polymeric layer. For the PEO/Zn@CS/DMC group, demeclocycline hydrochloride (C_21_H_21_ClN_2_O_8_·HCl, ≥99% purity; Sigma-Aldrich, USA) was incorporated into the CS solution^25^ to achieve a final quantity of 500 μg in the coating. To ensure precise dosing, the initial CS-antibiotic solution was prepared at a higher concentration of 3125 μg/mL. This concentration was selected to control the total amount of antibiotic deposited onto the surface during the coating process. Specifically, 80 μL aliquots of this concentrated solution were applied per layer. At 3125 μg/mL, each 80 μL aliquot contained 250 μg of DMC. The coating procedure involved two sequential applications of this aliquot, allowing the solvent to evaporate between layers to form a uniform film. After the second application, the expected cumulative demeclocycline content in the final CS film was 500 μg. This concentration was determined based on the minimum bactericidal concentration (MBC) results and was selected to achieve a balance between antimicrobial efficacy and cytocompatibility. Following preparation, all coated disks were stored in sealed 24-well plates wrapped in aluminum foil to prevent light exposure and subsequently placed in a desiccator to preserve the structural and chemical integrity of the polymeric layers until further characterization and analysis.

### Coatings Characterization

#### Surface Morphology and Topography

Surface morphology was examined using scanning electron microscopy (SEM) (JEOL JSM-601LA, Peabody, MA, USA), operating at an accelerating voltage of 15.0 kV and at a 500× magnification (*n* = 2/group). Cross-sectional morphology was assessed by embedding samples (n = 3/group) in poly­(methyl methacrylate) (PMMA) resin, followed by sectioning with a diamond-coated hard-tissue microtome (Leica Microsystems SP 1600, Nussloch, Germany). Coating thickness was quantified by measuring five randomly selected regions in SEM cross-sectional micrographs acquired at 2000× magnification using the ImageJ software. For three-dimensional surface characterization, a non-contact confocal laser scanning microscope (CLSM) (VK-X200 series, Keyence, Osaka, Japan) was employed. These images were subsequently processed and analyzed using the VK-Analyzer software program (Version 3.3.0.0, Keyence, Osaka, Japan) (*n* = 3/group).^73^

#### Roughness and Wettability

Surface roughness was evaluated by quantifying the average surface roughness (Ra). A contact profilometer (Dektak D150; Veeco, Plainview, NY, USA) was employed for these measurements, operating with a 0.25 mm cut-off length and a scanning speed of 0.05 mm/s over a 12-second acquisition period. To ensure representative surface characterization, three independent measurements were performed in randomly selected regions of each sample (*n* = 4/group).^31^ The surface wettability of the coatings was determined using the sessile drop technique. An automated goniometer (Ramé-Hart Instrument Co., Model 100-00, Succasunna, NJ, USA) was utilized to measure the static contact angle between the sample surfaces (*n* = 4/group) and a 10 μL droplet of deionized water. The acquired images were processed using DROPimage software (Ramé-Hart Instrument Co.).^73^

#### Chemical and Crystalline Composition

The elemental composition and atomic distribution of the coatings were assessed using energy-dispersive X-ray spectrometry (EDS; JEOL JSM-6010LA, Peabody, MA, USA). Analysis was conducted in three randomly selected regions per sample (*n* = 2/group). To further investigate the oxidation states of individual elements present in the coatings (*n* = 1/group), X-ray photoelectron spectroscopy (XPS; VSW HA100, Vacuum Science Workshop, Manchester, UK) was performed. The measurements were carried out using a spot size of 4 × 7 mm and an energy step size of 0.1 eV. Reference binding energies were obtained from the National Institute of Standards and Technology (NIST) XPS Online Database and corroborated with data from previous literature. The characterization of molecular bonds within the coatings (*n* = 1/group) was identified and confirmed using Fourier Transform Infrared Spectroscopy (FTIR; Jasco FTIR 410, Tokyo, Japan). Spectra acquisition was performed in the 4000–700 cm^–1^ frequency range, with a resolution of 4 cm^–1^ and an average of 128 scans. Crystalline phase identification (*n* = 1/group) was conducted using X-ray diffraction (XRD; Panalytical X’Pert3 Powder, Almelo, The Netherlands). The diffractometer operated with Cu–Kα radiation (λ = 1.5418 Å) at 45 kV and 40 mA, with a continuous scanning mode at a speed of 0.02°/s, enabling detailed structural analysis of the coating’s phase composition.^83^

#### UV–Vis Absorption Profile

UV–vis spectroscopy was employed to determine the absorption profile of demeclocycline within the blue-light visible spectrum. A solution of demeclocycline hydrochloride (500 μg/mL) was prepared in phosphate-buffered saline (PBS, pH 7.4) and analyzed within the wavelength range of 430–490 nm (*n* = 1). The measurements were performed using a UV–vis spectrophotometer (DU800, Beckman Coulter, Brea, CA, USA) operating in wavelength scan mode, enabling spectral acquisition across the defined range.

#### Photodynamic Characterization

The photodynamic activity of the coatings was assessed by monitoring the degradation kinetics of methylene blue (MB) under visible-light irradiation according to a previous report.^35^ Each sample was immersed in 2 mL of an aqueous MB solution (10 μmol/L) (P.A., CAS 61-73-4, Química Moderna, Barueri, SP, Brazil). The experiment was conducted under two independent conditions (*n* = 4/group): (i) samples kept in the dark (wrapped in foil) to serve as controls, and (ii) samples exposed to visible-light irradiation. The irradiation source consisted of a light-emitting diode (LED) lamp (CEFOP, SP, Brazil) with an emission spectrum of ∼450 nm. The lamp was positioned at a fixed distance of 5 cm from the sample using a utility clamp, and the light intensity reaching the surface was measured using a power meter (Ophir Optronics Solution Ltd., Jerusalem, Israel), registering an irradiance of approximately 95 mW/cm^2^. At predefined time intervals (15, 30, 60, 90, 120, 180, and 240 min), an aliquot of 200 μL was removed from each well following gentle homogenization of the solution to ensure representative sampling. The MB concentration in each aliquot was quantified by measuring absorbance at 664 nm using a microplate spectrophotometer (Multiskan Spectrum, Thermo Scientific, Waltham, MA, USA). After absorbance readings were obtained, the aliquots were returned to their respective wells to maintain the integrity of the degradation kinetics. The 24-well foil-wrapped plate was maintained in the same environmental conditions as the irradiated samples to ensure experimental consistency. The photodynamic activity of the coatings, expressed as the percentage of MB degradation, was calculated using the following equation:

$$photodynamic\;activity\;\left(\%\right)=\left[\frac{C_{0}-C}{C_{0}}\right]\times \left[\frac{C_{1}}{C_{0}}\right]\times 100$$

where *C*_0_ represents the initial MB concentration, *C* denotes the concentration at a given irradiation time *t*, and *C*_1_ corresponds to the MB concentration after the pre-adsorption step.^35^

#### ROS Generation

Reactive oxygen species generation was assessed using the 2′,7′-dichlorodihydrofluorescein diacetate (DCFH-DA) fluorescence assay (Invitrogen, Carlsbad, CA, USA). Because the assay was performed under cell-free conditions, DCFH-DA was chemically deacetylated before use. Briefly, 20 μL of a 10 mM DCFH-DA stock solution was mixed with 480 μL of 100 mM NaOH and incubated for 15 min at room temperature in the dark.^55^ The solution was then diluted to a final volume of 10 mL with 25 mM Tris buffer (pH 7.2), yielding a final DCFH concentration of 20 μM.^84^ The working solution was kept protected from light until use. The experimental groups included a negative control containing DCFH solution without a disk, PEO/Zn@CS, PEO/Zn@CS/DMC surfaces, and a DMC solution (500 μg/mL). Each condition was evaluated in quadruplicate under light and dark conditions. For the surface groups, one disk was placed in each well and incubated with 300 μL of the DCFH working solution. Samples assigned to the light condition were irradiated for 30 min using the same light source employed for DMC photoactivation, whereas dark controls were maintained for the same period under equivalent experimental conditions while protected from light. After incubation, the solutions were gently homogenized and immediately transferred to black 96-well microplates. Two technical aliquots of 150 μL were analyzed per experimental sample. Fluorescence intensity was measured at an excitation wavelength of 488 nm and an emission wavelength of 525 nm. Results were expressed as fluorescence intensity in arbitrary units (au).

### Mechanical Properties

#### Friction Coefficient, Wear Track Characterization, and Mass Loss

Tribological behavior, including friction coefficient, mass loss, and wear resistance, was investigated using a custom-built pin-on-disk tribometer developed at the Faculty of Mechanical Engineering, University of São Paulo, São Carlos, SP, Brazil. The counter body employed in the wear tests consisted of a 5 mm zirconia ball (Tosoh YTX Grinding Media), simulating articulation between the implant surface and potential antagonistic structures. The samples (*n* = 4/group) were immersed in SBF at 37 °C and pH 7.4 to replicate physiological conditions. The tribological tests were performed under the following parameters: a vertical load of 5 N, a track diameter of 7.5 mm, a sliding velocity of 0.01 m/s, and a total sliding duration of 100 seconds. A comprehensive description of the testing setup and methodology is available in previous literature.^31^ To determine the wear resistance of the coatings, the wear morphology and scar characteristics were analyzed via SEM (JEOL JSM-6010LA, Peabody, MA, USA). Additionally, wear track surface area was quantified using an optical microscope (VMM-100-BT; Walter UHL, Asslar, Germany) equipped with a high-resolution digital camera and an image analysis system (QC 220-HH QuadraCheck 200; Metronics Inc., Bedford, MA, USA). The total surface area of the wear tracks was determined by measuring the boundary edges of the scars. The mass loss (μg) was quantified by weighing the samples before (baseline) and after the tribological tests using a high-precision analytical balance (AUY-UNIBLOC, Shimadzu Corporation, Kyoto, Japan).^31^

#### Microhardness

The microhardness of the coatings (*n* = 4/group) was evaluated by using a Vickers microhardness tester (HMV-2, Shimadzu Corporation, Kyoto, Japan) equipped with a diamond indenter. The measurements were performed under a controlled load of 0.5 kgf, applied for 15 seconds to ensure a consistent indentation depth. Three randomly selected regions per sample were analyzed, and the Vickers hardness number (VHN) was calculated.^34^

#### DMC Release under Tribological Test

To assess the potential influence of mechanical wear on drug release kinetics, the release of demeclocycline from the PEO/Zn@CS/DMC group was quantified under tribological stress conditions. Following the wear test, the SBF in direct contact with the coated disks was collected for analysis. Two distinct conditions were evaluated: (i) SBF exposed to the tribological test, where the solution remained in contact with the surface while undergoing mechanical stress, and (ii) static SBF, where the solution was in contact with the coated disk for an equivalent duration (100 seconds) without applied mechanical loading. To quantify demeclocycline release, the collected SBF solutions were analyzed using spectrophotometry (DU800, Beckman Coulter, Brea, CA, USA). The demeclocycline concentration in μg/mL was determined based on a standard calibration curve, which exhibited a good linear correlation (*R* ^2^ = 0.9998) and was described by the regression equation *Y* = 0.008700*X* + 0.06726, where *Y* represents the absorbance and *X* the demeclocycline concentration.

#### Degradation and Release Profile

The degradation kinetics of the CS-based polymeric film were evaluated using SEM (JEOL JSM-6010LA, Peabody, MA, USA) to monitor morphological changes over time. To simulate *in vitro* degradation conditions, coated samples were immersed in PBS (pH 7.4) in the presence and absence of lysozyme (100 μg/mL; Sigma- Aldrich, St. Louis, MO, USA), the primary enzymatic agent responsible for CS degradation, and kept in the dark in an incubator at 37 °C. The degradation process was assessed at predefined time points of 1, 7, 14, 21, and 28 days of immersion, following previously established methodologies.^82,85^ At each time point, samples were retrieved from the incubation medium, air-dried, and sputter-coated with a thin layer of gold before SEM analysis. The release kinetics of DMC from the CS-based polymeric film were evaluated by spectrophotometric absorbance measurements at predetermined immersion intervals: 1, 6, 12 h, 1, 7, 14, 21, and 28 days. For this purpose, samples were immersed in 1 mL of PBS with or without lysozyme (100 μg/mL; Sigma-Aldrich, St. Louis, MO, USA) in 24-well plates to simulate enzymatic degradation. At each time point, the contents of each well were collected and analyzed spectrophotometrically. The absorbance measurements were performed using a microplate spectrophotometer (Multiskan, Thermo Scientific, Waltham, MA, USA). Immediately after spectrophotometric analysis, fresh PBS was added to each well to maintain the immersion conditions for subsequent time points. The DMC concentration in μg/mL was determined based on a standard calibration curve, which exhibited a strong linear correlation (*R* ^2^ = 0.9998) and was described by the regression equation *Y* = 0.008700*X* + 0.06726, where *Y* represents the absorbance and *X* the DMC concentration.

### Antimicrobial Analysis

#### Minimum Inhibitory Concentration and Minimum Bactericidal Concentration

To determine the optimal concentration of DMC for incorporation into the polymeric coating, the MIC and MBC assays were conducted using the standardized microdilution method.^86^ Saliva samples were collected from healthy volunteers at the Piracicaba Dental School—University of Campinas (UNICAMP) to obtain a complex polymicrobial inoculum. For this, whole stimulated saliva was collected from two healthy volunteers, who were informed of the study objectives and methodology and provided written informed consent, after approval by the Local Research Ethics Committee (CAAE: 74390923.7.0000.5418). Volunteers were required to fast for at least 1 h before collection and to have refrained from using antibiotics, antifungals, oral antiseptics, or any medication that could alter salivary flow for at least three months prior to the experiment. The collected saliva was incubated in a sterile, supplemented culture medium consisting of 80% modified fluid universal medium (mFUM), 67 mmol/L Sorensen buffer (pH 7.2), 10% brain heart infusion (BHI) broth (Difco Laboratories), and 10% sucrose solution. The inoculum was spectrophotometrically standardized to an optical density (OD) of 1 ± 0.02 at 550 nm using a spectrophotometer (DU800, Beckman Coulter), corresponding to an estimated bacterial concentration of 10^8^ CFU/mL.^62^ A 90 μL aliquot of the culture medium was added to a 10 μL aliquot of the standardized inoculum, and then transferred to each well of a 96-well microplate containing serial dilutions of DMC (Sigma-Aldrich, St. Louis, MO, USA) diluted in mFUM. The microplates were incubated at 37 °C for 24 h to allow bacterial growth in the presence of the antibiotic. The negative control consisted of the culture medium supplemented with a microbial inoculum but without any antimicrobial agent, serving as a reference for unrestricted microbial growth. In contrast, the positive control included the same culture medium and microbial inoculum but was supplemented with a 0.2% chlorhexidine solution. Bacterial metabolism was assessed using the resazurin reduction assay.^87^ After incubation, 30 μL of a 0.02% resazurin solution was added to each well, and the microplates were incubated for an additional 2 h at 37 °C.^87^ The MIC was defined as the lowest concentration of DMC that completely inhibited microbial growth, determined by the absence of a color change when compared to the negative control wells containing culture medium without bacterial inoculation.^48^ To determine the MBC, 10 μL aliquots were taken from wells corresponding to the MIC and higher concentrations and plated onto Columbia Blood Agar (CBA) supplemented with 5% defibrinated sheep blood. The plates were incubated at 37 °C for 48 h, and the MBC was defined as the lowest concentration of DMC that resulted in no detectable bacterial colony formation after the incubation period.^48^

### Biofilm Formation

#### Acquired Pellicle Formation

For biofilm microbiological analysis, disks from control and experimental groups were first submitted to the formation of an acquired pellicle formation. Saliva was collected fresh on the day of the experiment in 50 mL polypropylene tubes, following the same protocol and criteria described in the previous section. The samples were centrifuged at 10,000*g* for 10 min at 4 °C, and the supernatant was sterilized by filtration through a 0.22 μm membrane. For acquired pellicle formation, titanium disks were placed in 24-well polystyrene culture plates and immersed in 1 mL of the processed saliva for 30 min at 37 °C under gentle agitation (60 rpm).^62^

#### Biofilm Growth Conditions

Following acquired pellicle formation, biofilms were cultivated over a four-day period under two distinct growth phases to mimic the transition from aerobic to anaerobic biofilm microenvironment conditions. Initially, samples were randomly assigned to 24-well culture plates, with each well receiving 100 μL of unfiltered human saliva (inoculum) and 900 μL of a supplemented culture medium composed of 80% modified fluid universal medium (mFUM), 67 mmol/L Sorensen buffer (pH 7.2), 10% brain heart infusion (BHI) broth (Difco Laboratories), and 10% sucrose solution. The inoculum was standardized to an optical density (OD) of 1 ± 0.02 at 550 nm by spectrophotometry, corresponding to an estimated bacterial concentration of 10^8^ CFU/mL.^62^ The first phase, designed to facilitate bacterial adhesion and initial colonization, involved incubation at 37°C under 10% CO_2_ for 24 h. Following this adhesion period, the biofilms were washed with 0.9% NaCl to remove non-adherent cells and subsequently exposed to fresh mFUM medium to support biofilm maturation and biomass development. After 24 h, the biofilms underwent a second wash with 0.9% NaCl and were transferred to new 24-well plates, in which each well was replenished with 900 μL of fresh culture medium and 100 μL of newly collected and standardized human saliva inoculum. The samples were then incubated under strict anaerobic conditions (10% H_2_, 5% CO_2_, 85% N_2_, 37°C) for an additional 24 h. This second phase was specifically designed to favor the growth of anaerobic bacteria. The process was repeated, with biofilms being washed and reintroduced to fresh culture medium every 24 h, maintaining anaerobic conditions throughout the experiment until the completion of the 96-hour biofilm formation period.^62^

#### Photodynamic Antimicrobial Assay

To evaluate the antimicrobial efficacy of aPDT using blue light and DMC-loaded coatings, both control and experimental samples were placed in 24-well plates, each containing 1 mL of 0.9% NaCl. To prevent unintended light exposure, the plates were wrapped in aluminum foil prior to irradiation. Subsequently, samples were exposed to blue light (λ ≈ 450 nm) from an LED source for 30 min.^16,17^ The light source was maintained at a fixed distance of 5 cm from the sample to ensure uniform light exposure across all treated surfaces. To differentiate the photodynamic antimicrobial effects from any potential non-light-mediated antimicrobial activity, groups were maintained under identical conditions without irradiation, remaining wrapped in aluminum foil throughout the treatment. This approach ensured that any reductions in microbial viability observed in the light-exposed groups could be specifically attributed to the synergistic effect of blue light and DMC, rather than external factors such as environmental exposure or prolonged handling outside the incubator.

#### Bacterial Viability Analysis by Colony-Forming Unit (CFU) Counting

Following exposure to light or not, the disks with biofilms (*n* = 6/group) were transferred to cryogenic tubes containing 1 mL of 0.9% NaCl and subjected to sonication (7 W, 30 s) using a sonicator (S 150D, Branson Ultrasonics Corp.) to detach bacterial cells from the surface.^35^ A 100 μL aliquot of the sonicated suspension was serially diluted in 900 μL of 0.9% NaCl, and 10 μL of each dilution was plated in duplicate onto CBA supplemented with 5% defibrinated sheep blood for total bacterial count assessment. The CBA plates were incubated at anaerobic conditions (10% H_2_, 5% CO_2_, 85% N_2_, 37 °C) for 48 h. CFUs were quantified using a stereomicroscope, and the results were expressed as logarithmic CFU values per mL (log_10_ CFU/mL).^35^ To provide a qualitative visualization of microbial growth, a 50 μL aliquot of the sonicated biofilm suspension was spread onto CBA with 5% defibrinated sheep blood using a sterile cotton swab. After incubation under the respective experimental conditions, representative images of the plates were obtained to illustrate differences in colony density among the treatment groups.

#### Biofilm Metabolic Activity

Biofilm metabolic activity was evaluated using the XTT reduction assay, a colorimetric method based on the metabolic conversion of 2,3-bis­(2-methoxy-4-nitro-5-sulfophenyl)-2H-tetrazolium-5-carboxanilide (XTT) into a water-soluble formazan product by metabolically active cells. The XTT reagent (Sigma-Aldrich, St. Louis, MO, USA) was prepared and kept in contact with the microbial suspension according to a previously published protocol. The intensity of the colorimetric reaction, proportional to biofilm metabolic activity, was quantified by measuring absorbance at 492 nm using a UV–visible spectrophotometer (DU800, Beckman Coulter, Inc., Brea, CA, USA).^34^

#### Matrix Protein Content

Following sonication, a 200 μL aliquot of the biofilm suspension from samples exposed or not to light was transferred to a microcentrifuge tube containing 200 μL of 0.2 M sodium hydroxide (NaOH) and 2 mM EDTA.^88^ The samples were maintained at 4 °C under intermittent vortex agitation every 10 min for a total duration of 60 min to enhance protein solubilization. After this incubation period, the microtubes were centrifuged at 13,000*g* for 5 min at 4 °C. The supernatant, which contained the extracted extracellular proteins, was carefully collected and stored for further quantification. The total protein concentration in the extracellular matrix was determined using the bicinchoninic acid (BCA) assay (BCA Kit, Sigma-Aldrich, St. Louis, MO, USA), with bovine serum albumin as the standard protein.^89^ For quantification, 150 μL of the extracted protein suspension was transferred to a 96-well microplate and mixed with 150 μL of the working reagent, composed of Reagent A and Reagent B, following the manufacturer’s instructions. The microplate was incubated at 60 °C for 1 h to allow the colorimetric reaction to develop. Absorbance was subsequently measured at 562 nm using a microplate spectrophotometer (Multiskan, Thermo Scientific, Waltham, MA, USA). The protein concentration in each sample was calculated based on a standard curve prepared with bovine serum albumin at known concentrations.

#### Biofilm Structural Analysis by SEM and CLSM

The morphological and three-dimensional architecture of biofilms formed on the control and experimental surfaces were qualitatively analyzed using SEM and CLSM. For SEM analysis, biofilms were fixed in a 2.5% glutaraldehyde solution for 2 h, followed by sequential dehydration in graded ethanol baths (50, 60, 70, 90, and three 100% baths), each for 10 min. Samples were then air-dried at room temperature. Once dried, samples were mounted on stubs, sputter-coated with gold, and examined using a scanning electron microscope (JEOL JSM-5600LV) at an accelerating voltage of 15 kV.^30^ For CLSM analysis, biofilms were stained using the BacLight Live/Dead Viability Kit (Invitrogen-Molecular Probes). Live cells were stained green using SYTO-9 (excitation/emission: 480–500 nm), while dead cells were stained red using propidium iodide (excitation/emission: 490–635 nm).^34^ Samples were incubated in darkness for 20 min at 30 °C before imaging. A confocal inverted microscope (DMI 600 CS) coupled with a CLSM system (Leica Microsystems CMS) was used for image acquisition. Live and Dead fluorescence intensities were quantified using ImageJ software (NIH, Bethesda, MD, USA). Images were converted to 8-bit grayscale, thresholded to isolate fluorescent signals, and analyzed by particle analysis using a size range of 0–3 μm^2^. Integrated density was measured for each channel in each optical section. The relative percentages of viable and non-viable cells were calculated from the combined fluorescence intensity of the Live and Dead channels.

#### Microbial Composition by DNA–DNA Checkerboard Hybridization

The microbial composition of the polymicrobial biofilms was analyzed using the DNA–DNA checkerboard hybridization technique. Following biofilm formation and experimental treatments (with or without light exposure), biofilms were detached from the specimen surfaces and transferred into microcentrifuge tubes containing 150 μL of TE buffer (10 mM Tris-HCl and 1 mM EDTA, pH 7.6) and 100 μL of 0.5 M NaOH. For DNA denaturation, samples were boiled for 10 min and immediately neutralized with 0.8 mL of 5 M ammonium acetate. The released DNA was then loaded onto a Minislot 30 apparatus (Immunetics, Cambridge, MA, USA), allowing deposition into individual lanes. Hybridization was performed using a Miniblotter 45 system (Immunetics), in which the DNA samples were positioned perpendicular to the lanes containing DNA probes. Species-specific DNA probes targeting 40 bacterial taxa were labeled with digoxigenin and hybridized to the membranes at 42 °C for 20 h. Detection was carried out using an anti-digoxigenin antibody conjugated to alkaline phosphatase (Boehringer Mannheim, Indianapolis, IN, USA), followed by chemiluminescent substrate development. The emitted signal was captured, and bacterial counts were estimated semi-quantitatively by comparison with standards containing 10^5^ and 10^6^ bacterial cells. For each sample, total DNA probe counts and species-specific counts were determined. The relative abundance of each bacterial species was calculated as a percentage of the total detected signal. Additionally, microbial profiles were interpreted based on the microbial complexes described by Socransky et al., enabling ecological classification of the biofilm communities.^90,91^

#### Recolonization and Reusability Assay

To assess the reusability and recolonization potential of the experimental surfaces, a microcosm biofilm model was cultivated for 4 days using the previously described methodology.^62^ At the end of the biofilm formation period, disks from all experimental groups, including the PEO/Zn@CS/DMC group subjected to light exposure, were selected for further analysis. Since light exposure alone did not yield a significant antimicrobial effect in groups lacking DMC, only the PEO/Zn@CS/DMC + light condition was evaluated under photodynamic activation. After completing the four-day biofilm cultivation, the disks were carefully washed with 0.9% NaCl to remove non-adherent planktonic cells. Light-exposed samples underwent light irradiation, while dark-exposed samples remained wrapped in aluminum foil to prevent any unintended photodynamic activation. Following treatment, the disks were transferred to new 24-well culture plates, where they were re- exposed to a fresh microbial inoculum prepared from unfiltered human saliva and 900 μL of fresh culture medium (80% modified fluid universal medium and 10% BHI, supplemented with 10% sucrose). The re-inoculated samples were then incubated under anaerobic conditions (10% H_2_, 5% CO_2_, 85% N_2_, 37°C) to promote a second biofilm formation cycle for 24 h, simulating a reinfection scenario.^46^ Following this second biofilm formation period, the samples were carefully washed with 0.9% NaCl to remove non-adherent bacteria. The PEO/Zn@CS/DMC + Light group was again subjected to blue-light irradiation to assess whether repeated photodynamic activation could maintain its antimicrobial efficacy across multiple infection cycles. After the light exposure step, all samples were sonicated, serially diluted, and plated onto CBA supplemented with 5% sheep blood, as previously described.

### Biological Assays

#### Hydroxyapatite Formation

The ability of the coatings to induce hydroxyapatite formation was assessed according to an established protocol.^31^ Samples were immersed in SBF for 28 days under physiological conditions. Each sample was incubated in 10 mL of SBF per cm^2^ of surface area and maintained under static conditions at 37 ± 1 °C to replicate the ionic environment of human plasma. Following the immersion period, the samples were carefully rinsed with deionized water and air-dried to preserve the integrity of the mineral layer formed on the surface. Hydroxyapatite (HAp) deposition was analyzed using XRD to identify characteristic crystalline phases indicative of HAp formation, while SEM was employed to examine the morphological features of the mineralized layer.

#### Preosteoblastic Cell Culture and Viability

To evaluate the cytocompatibility of the experimental surfaces, pre-osteoblastic MC3T3-E1 cells, derived from mouse calvarial bone and obtained from a certified cell bank (ATCC CRL- 2594; Banco de Células do Rio de Janeiro), were cultured under indirect exposure to the experimental coatings. This approach followed the extract-based testing method, in which the eluates from each experimental group were collected and used as conditioned media for cell culture.^34^ Disks from each group were incubated with α-Minimum Essential Medium (α-MEM; Gibco, Life Technologies, USA), supplemented with 100 U/mL penicillin, 100 μg/mL streptomycin, and 10% fetal bovine serum (FBS; Gibco, Life Technologies, Grand Island, NY, USA), under controlled conditions of 5% CO_2_ and 37 °C. After 24 h of incubation, the culture medium in direct contact with the disks was collected and filtered through a 0.22 μm membrane to remove potential contaminants before being used for indirect cytotoxicity testing. MC3T3-E1 cells were seeded into 48-well polystyrene culture plates at a density of 1 × 10^4^ cells per well and maintained under standard conditions. After 24 h of cell adhesion, the culture medium was replaced with the collected eluates from each experimental group to simulate the effects of released components on cell metabolism. The working concentration was determined by measuring the 50% toxicity value (IC50) after dissolving increasing concentrations of DMC in the culture medium.^92^ The tested concentration (625 to 6 μg/mL) was selected based on the MIC and MBC. The metabolic activity of MC3T3-E1 cells was assessed using the alamarBlue assay after 1 day of incubation. After culture, the culture medium was aspirated, and 500 μL of fresh medium supplemented with 10% alamarBlue reagent (Invitrogen, Carlsbad, CA, USA) was added to each well. The plates were incubated at 37 °C for 4 h to allow viable cells to metabolically reduce resazurin to its fluorescent resorufin form. After incubation, 100 μL of the solution from each well was transferred to a 96-well plate for absorbance measurements at 570 and 600 nm using a microplate spectrophotometer (Multiskan, Thermo Scientific, Vantaa, Finland). The percentage of cell viability was calculated relative to the untreated cpTi control group.^34^

### Proteomic Profile

#### Protein Adsorption and Quantification

After approval by the local Research Ethics Committee (CAAE: 74390923.7.0000.5418), human blood plasma was used as the protein source to simulate the initial exposure of implant surfaces to blood components from the surgical bed. Disks from the control and experimental groups were individually incubated with 1 mL of human blood plasma under orbital agitation (75 rpm) at 37 °C for 2 h to allow protein adsorption onto the surface. After incubation, the specimens (*n* = 4/group) were gently rinsed twice with phosphate-buffered saline (PBS) to remove loosely bound and nonadherent proteins. The disks were then transferred to cryogenic tubes containing 1 mL of PBS, and the adsorbed protein layer was detached by sonication using a Cup Horn sonicator (5.5-in. cup, Q500, Qsonica, Newtown, CT, USA) operated at 80% amplitude for 60 s. The recovered protein suspensions were initially quantified using the bicinchoninic acid (BCA) assay. Fresh working reagent was prepared according to the manufacturer’s instructions (BCA Kit, Sigma-Aldrich, St. Louis, MO, USA), added to the samples, and incubated for 1 h at 60 °C in the dark.^34^ Protein concentration was calculated from a standard curve prepared with bovine serum albumin (BSA), and absorbance was measured at 562 nm using a microplate reader (Multiskan, Thermo Scientific, Vantaa, Finland). This quantification step was used to normalize the protein input for subsequent electrophoretic separation.

#### SDS-PAGE

To reduce albumin dominance and improve visualization of less abundant proteins adsorbed onto the surfaces, the extracted proteins were subsequently resolved by sodium dodecyl sulfate-polyacrylamide gel electrophoresis (SDS-PAGE). Approximately 10 μg of protein from each sample was dried in a vacuum concentrator, resuspended in 20 μL of 1× Laemmli sample buffer, heat-denatured at 95 °C for 5 min, briefly centrifuged, and allowed to cool to room temperature before loading. Protein separation was performed using hand-cast polyacrylamide gels consisting of a 12% resolving gel and a 5% stacking gel. After polymerization, the gels were mounted in the electrophoresis system and run in 1× running buffer. A molecular weight marker was loaded in the first lane, followed by the samples, and electrophoresis was carried out initially at 100 V until the dye front reached the interface between stacking and resolving gels, followed by 120 V until the dye front approached the bottom of the gel. Gels were then stained with Coomassie Brilliant Blue for 1 h at 37 °C under gentle agitation and destained with methanol/acetic acid-based destaining solution until clear band resolution was achieved.^93^

#### In Gel Digestion

After visualization, the gel region of interest was manually excised and cut into approximately 1 mm^3^ pieces for in-gel digestion. Gel pieces were transferred to low-bind microcentrifuge tubes and dehydrated twice with 200 μL acetonitrile for 10 min, followed by drying in a SpeedVac for 5–10 min after each dehydration step. Reduction was performed by adding 10 mM dithiothreitol (DTT) prepared in 25 mM ammonium bicarbonate (NH_4_HCO_3_), followed by incubation at 56 °C for 45–60 min. After cooling to room temperature, the reduction solution was removed, and cysteine residues were alkylated with 55 mM iodoacetamide (IAA) in 25 mM NH_4_HCO_3_ for 30 min at 37 °C in the dark. The gel pieces were then centrifuged, washed with 100 μL of 25 mM NH_4_HCO_3_, dehydrated again with 100 μL acetonitrile for 10 min, and dried in a SpeedVac. Proteolytic digestion was carried out by adding 10–15 μL of trypsin solution (12.5 ng/μL in 50 mM NH_4_HCO_3_), allowing enzyme absorption on ice for 30–45 min, and then adding 10–20 μL of 50 mM NH_4_HCO_3_ to fully submerge the gel fragments. Digestion proceeded overnight at 37 °C. Following digestion, the tubes were centrifuged, and the peptide-containing supernatant was collected into fresh low-bind tubes. Residual peptides were extracted from the gel matrix by three sequential incubations with 30 μL of 5% formic acid (FA) in 50% acetonitrile (ACN) for 20 min at room temperature, with centrifugation at 13,000 rpm for 10 min after each extraction. All extracts were pooled and concentrated in a SpeedVac to a final volume of approximately 5–10 μL without complete drying. Prior to mass spectrometric analysis, the peptide mixtures were desalted and purified using ZipTip C18 micropipette tips according to a standardized reverse-phase cleanup protocol.^93,94^

#### Mass Spectrometry

For proteomic analysis, purified peptide samples were analyzed by liquid chromatography coupled to high-resolution tandem mass spectrometry (LC-MS/MS) using a Thermo Scientific Orbitrap Q Exactive mass spectrometer equipped with an LC1000 system. Dried peptide samples were reconstituted in an LC-compatible solvent prior to injection, separated by reverse-phase liquid chromatography, ionized by electrospray ionization, and analyzed in data-dependent acquisition mode. The resulting MS and MS/MS spectra were used to characterize the protein profile adsorbed onto the control and experimental surfaces. Raw MS/MS spectra were processed and analyzed using the SEQUEST search engine implemented in Proteome Discoverer software (v3.1, Thermo Scientific). Spectral data were searched against a comprehensive human protein database (UniProtKB, including Swiss-Prot reviewed entries and TrEMBL unreviewed entries). Search parameters were defined as follows: precursor ion mass tolerance of 20 ppm and fragment ion mass tolerance of 50 ppm. Protein identification required at least two unique peptides per protein group to ensure high-confidence assignments. Label-free quantification was performed based on razor and unique peptides, with normalization to total peptide abundance across samples. All remaining parameters were maintained at default settings. Data validation was conducted at both peptide and protein levels using a false discovery rate (FDR) threshold of 1%, determined by a target-decoy strategy. Additionally, all identified peptides were manually inspected to ensure spectral quality. To comparatively assess the protein adsorption profile among experimental groups, protein lists obtained for each surface were analyzed using Venn diagrams generated with the InteractiveVenn platform. Protein–protein interaction (PPI) networks were constructed using the STRING database (Search Tool for the Retrieval of Interacting Genes/Proteins, version 12.0), considering both functional and physical associations. The interaction network was generated with the minimum required interaction score set to the highest confidence level (0.900). In the resulting networks, edges represent physical and functional protein–protein associations, with their thickness reflecting the strength and confidence of the supporting evidence. Nodes were clustered using k-means clustering. Functional enrichment analysis was performed using Gene Ontology (GO) annotations and Reactome pathway databases to characterize the biological significance of the adsorbed protein profiles. Visualization parameters were defined as follows: terms were sorted by signal strength, with a minimum signal threshold ≥1, and only terms with a false discovery rate (FDR) ≤0.05 were considered statistically significant. To reduce redundancy, enriched terms were grouped based on similarity, applying a similarity cutoff ≥0.5.

### Statistical Analysis

The sample size for each experimental test was determined using G*Power software (Heinrich Heine University, Düsseldorf, Germany) to ensure adequate statistical power based on pilot studies and previously published data. The calculations were conducted with a significance level (α) of 0.05 and a power (1−β) of 0.80. Following data collection, descriptive statistics were computed, including means and standard deviations for each experimental group. The Shapiro-Wilk test was applied to evaluate data normality. Homogeneity of variances was assessed using Levene’s test. To analyze the effects of surface treatment, a one-way analysis of variance (ANOVA) was performed, with surface modification serving as the independent variable. In cases where the assumption of homogeneity of variances was not met, Welch’s correction was applied to the ANOVA model to account for variance heterogeneity. Post hoc multiple comparisons were conducted using either Tukey’s or Games-Howell tests, allowing pairwise identification of significant differences among experimental groups. For comparisons of only two experimental conditions, Student’s *t* test was applied. All statistical analyses were performed using SPSS software (IBM Corp., Armonk, NY, USA), while graphical representations were generated using GraphPad Prism software (GraphPad Software, San Diego, CA, USA). A significance threshold of α = 0.05 was consistently applied across all tests to determine statistical significance.

## Supplementary Material



## Data Availability

The data supporting the findings of this study are available from the corresponding author upon reasonable request.

## Notes

Table of accession codes and protein names identified in the plasma-derived proteomic analysis; gene ontology biological process; and molecular function and reactome pathways enrichment analyses for PEO, PEO/Zn, and PEO/Zn@CS groups (PDF)
