# Supplementary material for: Photoresponsive Bilayer Coating Integrating a Porous Zinc Oxide Layer and a Chitosan–Antibiotic Drug Delivery System for Antimicrobial Photodynamic Therapy in Biomedical Implants
Source: ACS Mater Au. 2026 Oct 8;6(6):1464–94. doi: 10.1021/acsmaterialsau.6c00149 (PMC13760644; doi:10.1021/acsmaterialsau.6c00149)
Supplement: Supplementary file 1 [file mg6c00149_si_001.pdf]

Photoresponsive bilayer coating integrating a porous zinc oxide layer and chitosan-antibiotic drug delivery system for antimicrobial photodynamic therapy in biomedical implants

Samuel S. Malheiros[†], Maria Helena R. P. Borges[†], Elidiane C. Rangel[‡], Nilson C. Cruz[‡], Carlos A. Fortulan[§], Xi Rao[⁂], Liqun Xu[⁂], João Gabriel S. Souza[†⊥#], Eduardo B. Moffa[◊], Bruna E. Nagay[¥*], and Valentim A. R. Barão[†*]

[†] Department of Prosthodontics and Periodontology, Piracicaba Dental School, Universidade Estadual de Campinas (UNICAMP), Piracicaba, São Paulo 13414-903, Brazil.

[‡] Laboratory of Technological Plasmas, Institute of Science and Technology, São Paulo State University (UNESP), Sorocaba, São Paulo 18087-180, Brazil.

[§] Department of Mechanical Engineering, University of São Paulo (USP), São Carlos, São Paulo 13566-590, Brazil.

[⁂] BRICS Joint Laboratory on Biomedical Materials, School of Materials and Energy, Southwest University, Chongqing 400715, PR China.

[⊥] Dental Research Division, Universidade Guarulhos, Guarulhos, São Paulo 07023-070, Brazil.

[#] Faculdade Israelita de Ciências da Saúde Albert Einstein, Hospital Israelita Albert Einstein, São Paulo, São Paulo 05652-900, Brazil.

[◊] Department of Dentistry, Centro Universitário das Faculdades Associadas (UNIFAE), São João da Boa Vista, São Paulo 13870-377, Brazil.

[¥] Department of Prosthodontics and Periodontology, Bauru School of Dentistry, University of São Paulo (USP), Bauru, São Paulo 17012-901, Brazil.

* Valentim A. R. Barão and Bruna E. Nagay share the position of senior authors.

Corresponding authors:

E-mail: vbarao@unicamp.br (V.A.R.B.); bruna.nagay@usp.br (B.E.N.).

Table S 1. Protein accession number and protein names.

Protein Accessions	Protein name
**A0A024R6N5**	Serpin family A member 1
**A0A075B6H7**	Probable non-functional immunoglobulin kappa variable 3-7
**A0A075B6H9**	Immunoglobulin lambda variable 4-69
**A0A075B6I0**	Immunoglobulin lambda variable 8-61
**A0A075B6I1**	Immunoglobulin lambda variable 4-60
**A0A075B6I4**	Immunoglobulin lambda variable 10-54
**A0A075B6I9**	Immunoglobulin lambda variable 7-46
**A0A075B6J9**	Immunoglobulin lambda variable 2-18
**A0A075B6K4**	Immunoglobulin lambda variable 3-10
**A0A075B6K5**	Immunoglobulin lambda variable 3-9
**A0A075B6P5**	Immunoglobulin kappa variable 2-28
**A0A075B6R2**	Immunoglobulin heavy variable 4-4
**A0A075B6R9**	Probable non-functional immunoglobulin kappa variable 2D-24
**A0A075B6S2**	Immunoglobulin kappa variable 2D-29
**A0A075B6S5**	Immunoglobulin kappa variable 1-27
**A0A075B7B8**	Immunoglobulin heavy variable 3/OR16-12 (non-functional) (Fragment)
**A0A087WSX0**	Immunoglobulin lambda variable 5-45
**A0A087WSY4**	Immunoglobulin heavy variable 4-30-2
**A0A087WSY5**	Carboxypeptidase B2
**A0A087WSY6**	Immunoglobulin kappa variable 3D-15
**A0A087WT04**	PMF1-BGLAP readthrough
**A0A087WT21**	CECR2 histone acetyl-lysine reader
**A0A087WT59**	Transthyretin
**A0A087WV42**	Proline rich protein HaeIII subfamily 1
**A0A087WVQ9**	Elongation factor 1-alpha
**A0A087WWU8**	Tropomyosin 3
**A0A087WXI2**	Fc gamma binding protein
**A0A087X295**	tRNA (34-2'-O)-methyltransferase regulator WDR6
**A0A096LPE2**	SAA2-SAA4 readthrough
**A0A0A0MRJ7**	Coagulation factor V
**A0A0A0MRZ8**	Immunoglobulin kappa variable 3D-11
**A0A0A0MS09**	Immunoglobulin heavy constant delta (Fragment)
**A0A0A0MS14**	Immunoglobulin heavy variable 1-45
**A0A0A0MS15**	Immunoglobulin heavy variable 3-49
**A0A0A0MSI0**	Peroxiredoxin-1 (Fragment)
**A0A0A0MSJ8**	Protein phosphatase 6 regulatory subunit 2 (Fragment)
**A0A0A0MSV6**	Complement C1q B chain (Fragment)
**A0A0A0MSY7**	Thymidine kinase 2, mitochondrial
**A0A0A0MT36**	Immunoglobulin kappa variable 6D-21
**A0A0A0MT69**	Immunoglobulin kappa joining 4 (Fragment)
**A0A0A0MT89**	Immunoglobulin kappa joining 1
**A0A0B4J1U3**	Immunoglobulin lambda variable 1-36
**A0A0B4J1U7**	Immunoglobulin heavy variable 6-1
**A0A0B4J1V0**	Immunoglobulin heavy variable 3-15
**A0A0B4J1V6**	Immunoglobulin heavy variable 3-73
**A0A0B4J1X5**	Immunoglobulin heavy variable 3-74
**A0A0B4J1X8**	Immunoglobulin heavy variable 3-43

**A0A0B4J1Y8**	Immunoglobulin lambda variable 9-49
**A0A0B4J231**	Immunoglobulin lambda like polypeptide 5
**A0A0B4J259**	Lysozyme C
**A0A0B4J2B8**	Immunoglobulin heavy variable 1/OR15-9 (non-functional) (Fragment)
**A0A0B4J2D9**	Immunoglobulin kappa variable 1D-13
**A0A0C4DFP6**	Cartilage acidic protein 1
**A0A0C4DG51**	Patatin like phospholipase domain containing 8 (Fragment)
**A0A0C4DGG0**	Family with sequence similarity 186 member B (Fragment)
**A0A0C4DGN4**	Zymogen granule protein 16B
**A0A0C4DH24**	Immunoglobulin kappa variable 6-21
**A0A0C4DH25**	Immunoglobulin kappa variable 3D-20
**A0A0C4DH29**	Immunoglobulin heavy variable 1-3
**A0A0C4DH31**	Immunoglobulin heavy variable 1-18
**A0A0C4DH34**	Immunoglobulin heavy variable 4-28
**A0A0C4DH35**	Probable non-functional immunoglobulin heavy variable 3-35
**A0A0C4DH36**	Probable non-functional immunoglobulin heavy variable 3-38
**A0A0C4DH38**	Immunoglobulin heavy variable 5-51
**A0A0C4DH41**	Immunoglobulin heavy variable 4-61
**A0A0C4DH43**	Immunoglobulin heavy variable 2-70D
**A0A0C4DH67**	Immunoglobulin kappa variable 1-8
**A0A0C4DH73**	Immunoglobulin kappa variable 1-12
**A0A0G2JHN4**	Coiled-coil alpha-helical rod protein 1 (Fragment)
**A0A0G2JNB4**	Proline rich protein BstNI subfamily 3
**A0A0G2JRQ6**	Ig-like domain-containing protein (Fragment)
**A0A0J9YW62**	Immunoglobulin V-set domain-containing protein (Fragment)
**A0A0J9YX35**	Immunoglobulin heavy variable 3-64D
**A0A0J9YXX1**	Immunoglobulin heavy variable 5-10-1
**A0A0J9YY99**	Ig-like domain-containing protein (Fragment)
**A0A0S2Z4L3**	Vitamin K-dependent protein S (Fragment)
**A0A0U1RR32**	Histone H2A
**A0A0U1RR70**	non-specific serine/threonine protein kinase
**A0A182DWH7**	Selenoprotein P (Fragment)
**A0A1B0GV23**	Cathepsin D
**A0A1B0GVI3**	Keratin, type I cytoskeletal 10
**A0A1W2PP30**	FLII actin remodeling protein (Fragment)
**A0A2R8Y4R7**	Mitochondrial tRNA-specific 2-thiouridylase 1 (Fragment)
**A0A3B3IRL2**	Cellular repressor of E1A stimulated genes 1
**A0A3B3ISG5**	Insulin degrading enzyme
**A0A3B3ISR2**	complement subcomponent C1r
**A0A3B3ISU0**	Desmocollin 2
**A0A494C0J7**	Transglutaminase-like domain-containing protein
**A0A4W8ZXM2**	Immunoglobulin heavy variable 3-72
**A0A590UIX5**	Deleted in malignant brain tumors 1 (Fragment)
**A0A590UJJ6**	Histone H3
**A0A5F9ZH82**	Lysosomal acid phosphatase (Fragment)
**A0A5H1ZRQ7**	Immunoglobulin lambda constant 7 (Fragment)
**A0A6Q8PFJ0**	Lamin A/C
**A0A6Q8PFK8**	Heat shock protein family B (small) member 1

**A0A7I2V2D2**	Serpin family G member 1
**A0A7I2V2J1**	Cathepsin B
**A0A7I2V2Q8**	Dipeptidyl peptidase 1
**A0A7I2V3A9**	Kinesin-like protein
**A0A7I2V5U0**	ATP binding cassette subfamily A member 1
**A0A7I2YQQ3**	folate gamma-glutamyl hydrolase
**A0A7P0T8D1**	Angiotensinogen
**A0A7P0TAB0**	non-specific serine/threonine protein kinase
**A0A804F6T5**	Pyruvate kinase
**A0A804HIA4**	Forkhead associated phosphopeptide binding domain 1
**A0A8I5KSC3**	Neurofibromin 1
**A0A8Q3SI22**	Complement C3 (Fragment)
**A0A8Q3SI37**	Complement component C9
**A0A8Q3SI99**	Complement factor H
**A0A8Q3SIA1**	Sushi domain-containing protein
**A0A8Q3SID6**	Complement C5
**A0A8Q3SIM0**	Complement C7
**A0A8Q3SIZ0**	Complement C1q C chain
**A0A8Q3WKT7**	Complement C8 alpha chain
**A0A8Q3WKW0**	Complement factor D
**A0A8Q3WL25**	Coagulation factor XII
**A0A8Q3WL56**	Complement component C8 beta chain
**A0A8V8TN80**	Low affinity immunoglobulin gamma Fc region receptor III-A (Fragment)
**A0A994J439**	Inter-alpha-trypsin inhibitor heavy chain H3
**A0A9H3ZR93**	Immunoglobulin heavy constant gamma 3 (G3m marker) (Fragment)
**A0A9L9PXQ4**	Lysosomal associated membrane protein 2
**A0AAG2SXZ4**	Ig-like domain-containing protein
**A0AAG2T928**	Immunoglobulin kappa variable 1-33
**A0AAG2TV02**	HCG2003024
**A0AAG2TVE0**	Ig-like domain-containing protein
**A0AAG2UUW0**	Apolipoprotein B
**A2NJV5**	Immunoglobulin kappa variable 2-29
**A6NNK5**	Tumor protein p53 binding protein 1
**A6PVI2**	Plasminogen
**A6XGL3**	Protease serine 1
**B0YIW2**	Apolipoprotein C-III
**B1AH94**	Apolipoprotein L1
**B1AHL2**	Fibulin-1
**B1AKG0**	Complement factor H related 1
**B1AN48**	Small proline rich protein 3 (Fragment)
**B4DEV8**	Proteasome subunit alpha type
**B4DV12**	Ubiquitin B
**B4E1Z4**	Complement C2
**B7Z5J4**	Carboxypeptidase A4
**C9IZP8**	Complement C1s (Fragment)
**C9JEE0**	Immunoglobulin lambda like polypeptide 1 (Fragment)
**C9JF17**	Apolipoprotein D (Fragment)
**C9JPQ9**	Fibrinogen gamma chain (Fragment)

**C9JQK2**	Ankyrin repeat domain 53
**D6RAR4**	HGF activator
**D6RD17**	Joining chain of multimeric IgA and IgM (Fragment)
**D6RF35**	Vitamin D-binding protein
**D6RHX1**	Mucin 7, secreted (Fragment)
**E5RIJ0**	DNA polymerase
**E7END6**	Vitamin K-dependent protein C
**E7EQB2**	Lactotransferrin (Fragment)
**E7EQL5**	Dynein cytoplasmic 1 intermediate chain 2 (Fragment)
**E7ETH0**	Complement factor I
**E7EX29**	14-3-3 protein zeta/delta (Fragment)
**E7EXA8**	Proline rich protein BstNI subfamily 4
**E9PEK4**	receptor protein-tyrosine kinase
**E9PHK0**	C-type lectin domain family 3 member B
**E9PHY8**	Maestro heat like repeat family member 1
**E9PKE3**	Heat shock cognate 71 kDa protein
**F2Z3N2**	Complement C2
**F5H5D3**	Tubulin alpha chain
**F5H5U2**	ATP-dependent RNA helicase
**F8VV13**	Mucin like 1
**F8W876**	MBL associated serine protease 1
**F8WD03**	HYDIN axonemal central pair apparatus protein
**G3V2W1**	Serpin family A member 10
**G3V4T5**	Eukaryotic translation initiation factor 2 subunit 1 (Fragment)
**G3XAK1**	Macrophage stimulating 1
**G3XAP6**	Cartilage oligomeric matrix protein
**H0Y5H9**	Serpin family B member 4 (Fragment)
**H0Y8L3**	Transforming growth factor beta induced (Fragment)
**H0YAC1**	Uncharacterized protein (Fragment)
**H0YI52**	RNA helicase (Fragment)
**H0YMW4**	Annexin
**H3BQN4**	Fructose-bisphosphate aldolase
**H3BRJ9**	Cholesteryl ester transfer protein
**I3L145**	Sex hormone-binding globulin
**J3QS61**	Oxysterol binding protein like 1A
**J3QSF7**	Myeloperoxidase (Fragment)
**K7EMD9**	Keratin 13 (Fragment)
**K7ER74**	Apolipoprotein C-II
**K7ERI9**	Apolipoprotein C-I (Fragment)
**M0QYU8**	Kallikrein related peptidase 7 (Fragment)
**M0R104**	Hypoxia inducible factor 3 subunit alpha
**O00391**	Sulfhydryl oxidase 1
**O15230**	Laminin subunit alpha-5
**O43790**	Keratin, type II cuticular Hb6
**O43866**	CD5 antigen-like
**O60287**	Nucleolar pre-ribosomal-associated protein 1
**O60814**	Histone H2B type 1-K
**O75223**	Gamma-glutamylcyclotransferase

**O75635**	Serpin B7
**O75636**	Ficolin-3
**O75882**	Attractin
**O75884**	Serine hydrolase RBBP9
**O94885**	SAM and SH3 domain-containing protein 1
**O95147**	Dual specificity protein phosphatase 14
**O95445**	Apolipoprotein M
**O95497**	Pantetheinase
**P00450**	Ceruloplasmin
**P00488**	Coagulation factor XIII A chain
**P00734**	Prothrombin
**P00738**	Haptoglobin
**P00739**	Haptoglobin-related protein
**P00740**	Coagulation factor IX
**P00742**	Coagulation factor X
**P01008**	Antithrombin-III
**P01009**	Alpha-1-antitrypsin
**P01011**	Alpha-1-antichymotrypsin
**P01023**	Alpha-2-macroglobulin
**P01024**	Complement C3
**P01036**	Cystatin-S
**P01040**	Cystatin-A
**P01042**	Kininogen-1
**P01597**	Immunoglobulin kappa variable 1-39
**P01599**	Immunoglobulin kappa variable 1-17
**P01601**	Immunoglobulin kappa variable 1D-16
**P01602**	Immunoglobulin kappa variable 1-5
**P01619**	Immunoglobulin kappa variable 3-20
**P01624**	Immunoglobulin kappa variable 3-15
**P01700**	Immunoglobulin lambda variable 1-47
**P01701**	Immunoglobulin lambda variable 1-51
**P01703**	Immunoglobulin lambda variable 1-40
**P01704**	Immunoglobulin lambda variable 2-14
**P01705**	Immunoglobulin lambda variable 2-23
**P01706**	Immunoglobulin lambda variable 2-11
**P01709**	Immunoglobulin lambda variable 2-8
**P01714**	Immunoglobulin lambda variable 3-19
**P01717**	Immunoglobulin lambda variable 3-25
**P01721**	Immunoglobulin lambda variable 6-57
**P01742**	Immunoglobulin heavy variable 1-69
**P01743**	Immunoglobulin heavy variable 1-46
**P01766**	Immunoglobulin heavy variable 3-13
**P01780**	Immunoglobulin heavy variable 3-7
**P01833**	Polymeric immunoglobulin receptor
**P01834**	Immunoglobulin kappa constant
**P01857**	Immunoglobulin heavy constant gamma 1
**P01859**	Immunoglobulin heavy constant gamma 2
**P01861**	Immunoglobulin heavy constant gamma 4

**P01871**	Immunoglobulin heavy constant mu
**P01876**	Immunoglobulin heavy constant alpha 1
**P01877**	Immunoglobulin heavy constant alpha 2
**P02042**	Hemoglobin subunit delta
**P02452**	Collagen alpha-1(I) chain
**P02533**	Keratin, type I cytoskeletal 14
**P02538**	Keratin, type II cytoskeletal 6A
**P02647**	Apolipoprotein A-I
**P02649**	Apolipoprotein E
**P02652**	Apolipoprotein A-II
**P02671**	Fibrinogen alpha chain
**P02675**	Fibrinogen beta chain
**P02679**	Fibrinogen gamma chain
**P02743**	Serum amyloid P-component
**P02745**	Complement C1q subcomponent subunit A
**P02749**	Beta-2-glycoprotein 1
**P02750**	Leucine-rich alpha-2-glycoprotein
**P02751**	Fibronectin
**P02753**	Retinol-binding protein 4
**P02760**	Protein AMBP
**P02763**	Alpha-1-acid glycoprotein 1
**P02765**	Alpha-2-HS-glycoprotein
**P02768**	Albumin
**P02787**	Serotransferrin
**P02790**	Hemopexin
**P04003**	C4b-binding protein alpha chain
**P04004**	Vitronectin
**P04040**	Catalase
**P04083**	Annexin A1
**P04180**	Phosphatidylcholine-sterol acyltransferase
**P04196**	Histidine-rich glycoprotein
**P04211**	Immunoglobulin lambda variable 7-43
**P04217**	Alpha-1B-glycoprotein
**P04259**	Keratin, type II cytoskeletal 6B
**P04264**	Keratin, type II cytoskeletal 1
**P04275**	von Willebrand factor
**P04406**	Glyceraldehyde-3-phosphate dehydrogenase
**P04430**	Immunoglobulin kappa variable 1-16
**P04746**	Pancreatic alpha-amylase
**P05089**	Arginase-1
**P05109**	Protein S100-A8
**P05120**	Plasminogen activator inhibitor 2
**P05154**	Plasma serine protease inhibitor
**P05160**	Coagulation factor XIII B chain
**P05543**	Thyroxine-binding globulin
**P05546**	Heparin cofactor 2
**P06276**	Cholinesterase
**P06310**	Immunoglobulin kappa variable 2-30

**P06312**	Immunoglobulin kappa variable 4-1
**P06396**	Gelsolin
**P06702**	Protein S100-A9
**P06703**	Protein S100-A6
**P06727**	Apolipoprotein A-IV
**P06733**	Alpha-enolase
**P07359**	Platelet glycoprotein Ib alpha chain
**P07360**	Complement component C8 gamma chain
**P08123**	Collagen alpha-2(I) chain
**P08185**	Corticosteroid-binding globulin
**P08246**	Neutrophil elastase
**P08519**	Apolipoprotein(a)
**P08571**	Monocyte differentiation antigen CD14
**P08697**	Alpha-2-antiplasmin
**P08727**	Keratin, type I cytoskeletal 19
**P08779**	Keratin, type I cytoskeletal 16
**P09211**	Glutathione S-transferase P
**P09871**	Complement C1s subcomponent
**P0C0L4**	Complement C4-A
**P0C0L5**	Complement C4-B
**P0DOY2**	Immunoglobulin lambda constant 2
**P0DP01**	Immunoglobulin heavy variable 1-8
**P0DP23**	Calmodulin-1
**P10599**	Thioredoxin
**P10909**	Clusterin
**P11021**	Endoplasmic reticulum chaperone BiP
**P11279**	Lysosome-associated membrane glycoprotein 1
**P12035**	Keratin, type II cytoskeletal 3
**P12273**	Prolactin-inducible protein
**P13639**	Elongation factor 2
**P13646**	Keratin, type I cytoskeletal 13
**P13647**	Keratin, type II cytoskeletal 5
**P13671**	Complement component C6
**P13796**	Plastin-2
**P14151**	L-selectin
**P14923**	Junction plakoglobin
**P15169**	Carboxypeptidase N catalytic chain
**P15814**	Immunoglobulin lambda-like polypeptide 1
**P15924**	Desmoplakin
**P17066**	Heat shock 70 kDa protein 6
**P18206**	Vinculin
**P18428**	Lipopolysaccharide-binding protein
**P19013**	Keratin, type II cytoskeletal 4
**P19652**	Alpha-1-acid glycoprotein 2
**P19823**	Inter-alpha-trypsin inhibitor heavy chain H2
**P19827**	Inter-alpha-trypsin inhibitor heavy chain H1
**P20618**	Proteasome subunit beta type-1
**P20742**	Pregnancy zone protein

**P20851**	C4b-binding protein beta chain
**P20930**	Filaggrin
**P22735**	Protein-glutamine gamma-glutamyltransferase K
**P22792**	Carboxypeptidase N subunit 2
**P22891**	Vitamin K-dependent protein Z
**P23083**	Immunoglobulin heavy variable 1-2
**P23142**	Fibulin-1
**P23280**	Carbonic anhydrase 6
**P23490**	Loricrin
**P25311**	Zinc-alpha-2-glycoprotein
**P27169**	Serum paraoxonase/arylesterase 1
**P27482**	Calmodulin-like protein 3
**P29508**	Serpin B3
**P29622**	Kallistatin
**P31025**	Lipocalin-1
**P31151**	Protein S100-A7
**P31944**	Caspase-14
**P31947**	14-3-3 protein sigma
**P31949**	Protein S100-A11
**P32119**	Peroxiredoxin-2
**P35527**	Keratin, type I cytoskeletal 9
**P35858**	Insulin-like growth factor-binding protein complex acid labile subunit
**P35908**	Keratin, type II cytoskeletal 2 epidermal
**P36955**	Pigment epithelium-derived factor
**P43251**	Biotinidase
**P43652**	Afamin
**P47929**	Galectin-7
**P48668**	Keratin, type II cytoskeletal 6C
**P51884**	Lumican
**P55058**	Phospholipid transfer protein
**P59665**	Neutrophil defensin 1
**P60014**	Keratin-associated protein 10-10
**P60174**	Triosephosphate isomerase
**P60709**	Actin, cytoplasmic 1
**P62805**	Histone H4
**P68871**	Hemoglobin subunit beta
**P69905**	Hemoglobin subunit alpha
**P80108**	Phosphatidylinositol-glycan-specific phospholipase D
**P80748**	Immunoglobulin lambda variable 3-21
**P81605**	Dermcidin
**Q01469**	Fatty acid-binding protein 5
**Q01546**	Keratin, type II cytoskeletal 2 oral
**Q02388**	Collagen alpha-1(VII) chain
**Q02413**	Desmoglein-1
**Q04695**	Keratin, type I cytoskeletal 17
**Q07283**	Trichohyalin
**Q07890**	Son of sevenless homolog 2
**Q08380**	Galectin-3-binding protein

**Q08554**	Desmocollin-1
**Q13296**	Mammaglobin-A
**Q13790**	Apolipoprotein F
**Q13835**	Plakophilin-1
**Q13867**	Bleomycin hydrolase
**Q13885**	Tubulin beta-2A chain
**Q14166**	Tubulin--tyrosine ligase-like protein 12
**Q14247**	Src substrate cortactin
**Q14376**	UDP-glucose 4-epimerase
**Q14520**	Hyaluronan-binding protein 2
**Q14574**	Desmocollin-3
**Q14624**	Inter-alpha-trypsin inhibitor heavy chain H4
**Q15323**	Keratin, type I cuticular Ha1
**Q15517**	Corneodesmosin
**Q15828**	Cystatin-M
**Q16610**	Extracellular matrix protein 1
**Q5D862**	Filaggrin-2
**Q5T749**	Keratinocyte proline-rich protein
**Q5T750**	Protein KPLCE
**Q6A162**	Keratin, type I cytoskeletal 40
**Q6E0U4**	Dermokine
**Q6EMK4**	Vasorin
**Q6KB66**	Keratin, type II cytoskeletal 80
**Q6UWP8**	Suprabasin
**Q6UX71**	Plexin domain-containing protein 2
**Q6ZVX7**	F-box only protein 50
**Q7L1S5**	Carbohydrate sulfotransferase 9
**Q7Z591**	Microtubule organization protein AKNA
**Q7Z794**	Keratin, type II cytoskeletal 1b
**Q86Y46**	Keratin, type II cytoskeletal 73
**Q86YZ3**	Hornerin
**Q8IW75**	Serpin A12
**Q8IWU2**	Serine/threonine-protein kinase LMTK2
**Q8N1N4**	Keratin, type II cytoskeletal 78
**Q8N4F0**	BPI fold-containing family B member 2
**Q8TCG2**	Phosphatidylinositol 4-kinase type 2-beta
**Q8TDL5**	BPI fold-containing family B member 1
**Q8WVV4**	Protein POF1B
**Q92764**	Keratin, type I cuticular Ha5
**Q96DR5**	BPI fold-containing family A member 2
**Q96N23**	Cilia- and flagella-associated protein 54
**Q96P63**	Serpin B12
**Q96PD5**	N-acetylmuramoyl-L-alanine amidase
**Q96QA5**	Gasdermin-A
**Q9BZQ2**	Testicular spindle-associated protein SHCBP1L
**Q9HC84**	Mucin-5B
**Q9HCS7**	Pre-mRNA-splicing factor SYF1
**Q9NP55**	BPI fold-containing family A member 1

**Q9NSB2**	Keratin, type II cuticular Hb4
**Q9NTI5**	Sister chromatid cohesion protein PDS5 homolog B
**Q9NZI7**	Upstream-binding protein 1
**Q9NZP8**	Complement C1r subcomponent-like protein
**Q9NZT1**	Calmodulin-like protein 5
**Q9UBB9**	Tuftelin-interacting protein 11
**Q9UGM5**	Fetuin-B

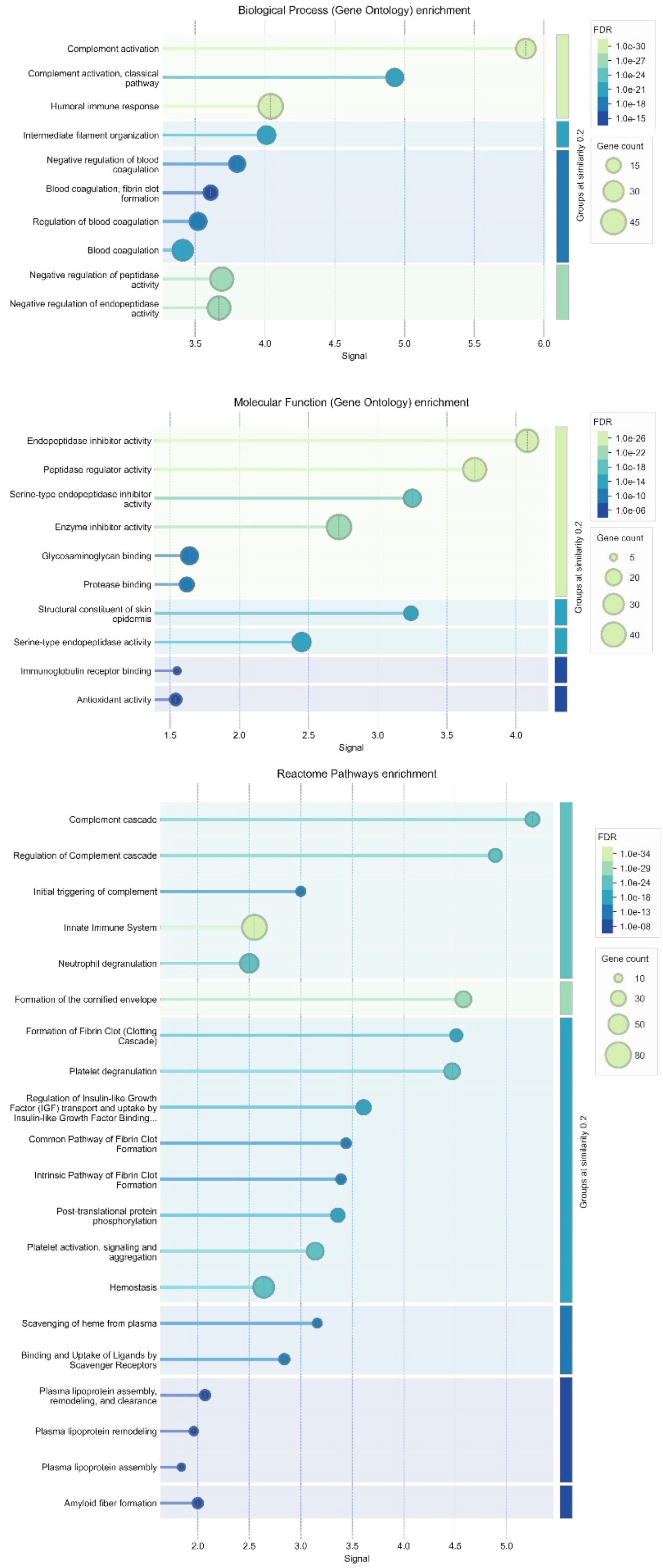

Figure S 1. PPI Network of proteins from plasma adsorbed on PEO surface.

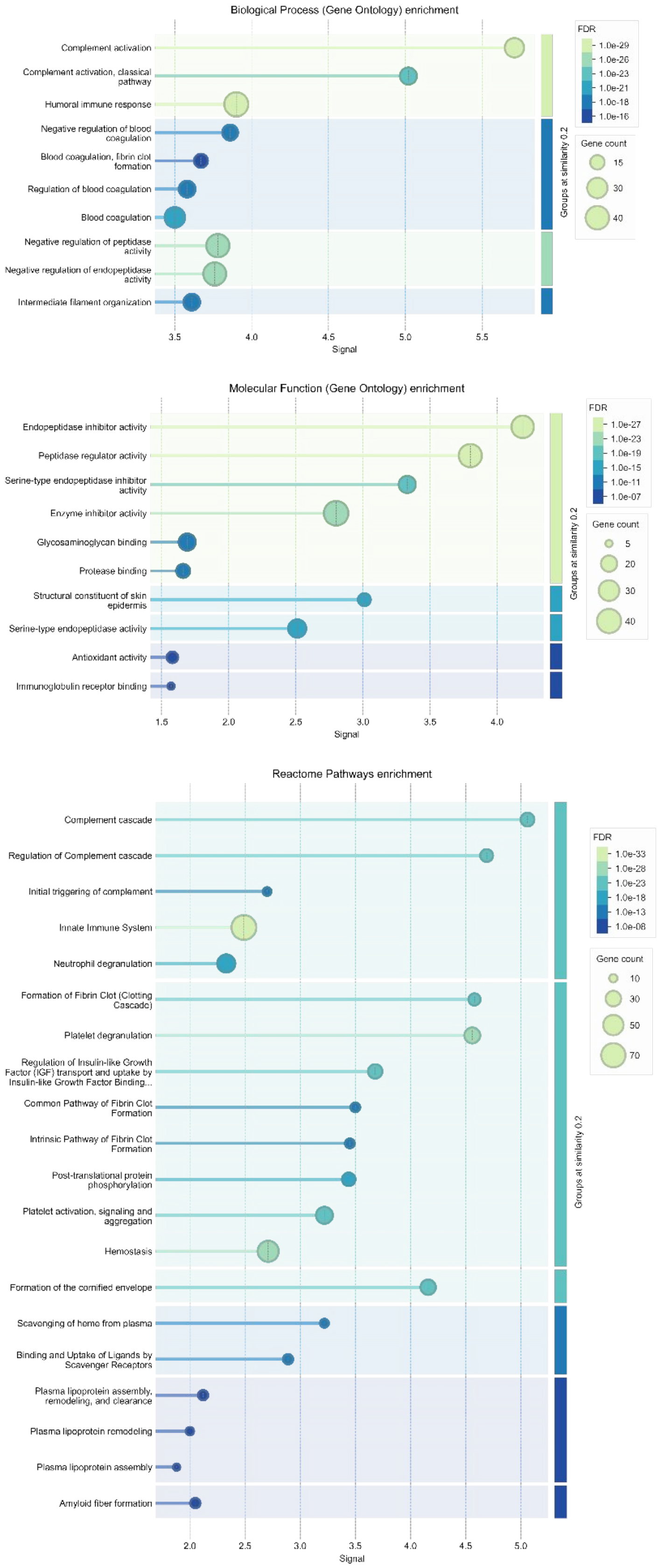

Figure S 2. PPI Network of proteins from plasma adsorbed on PEO/Zn surface.

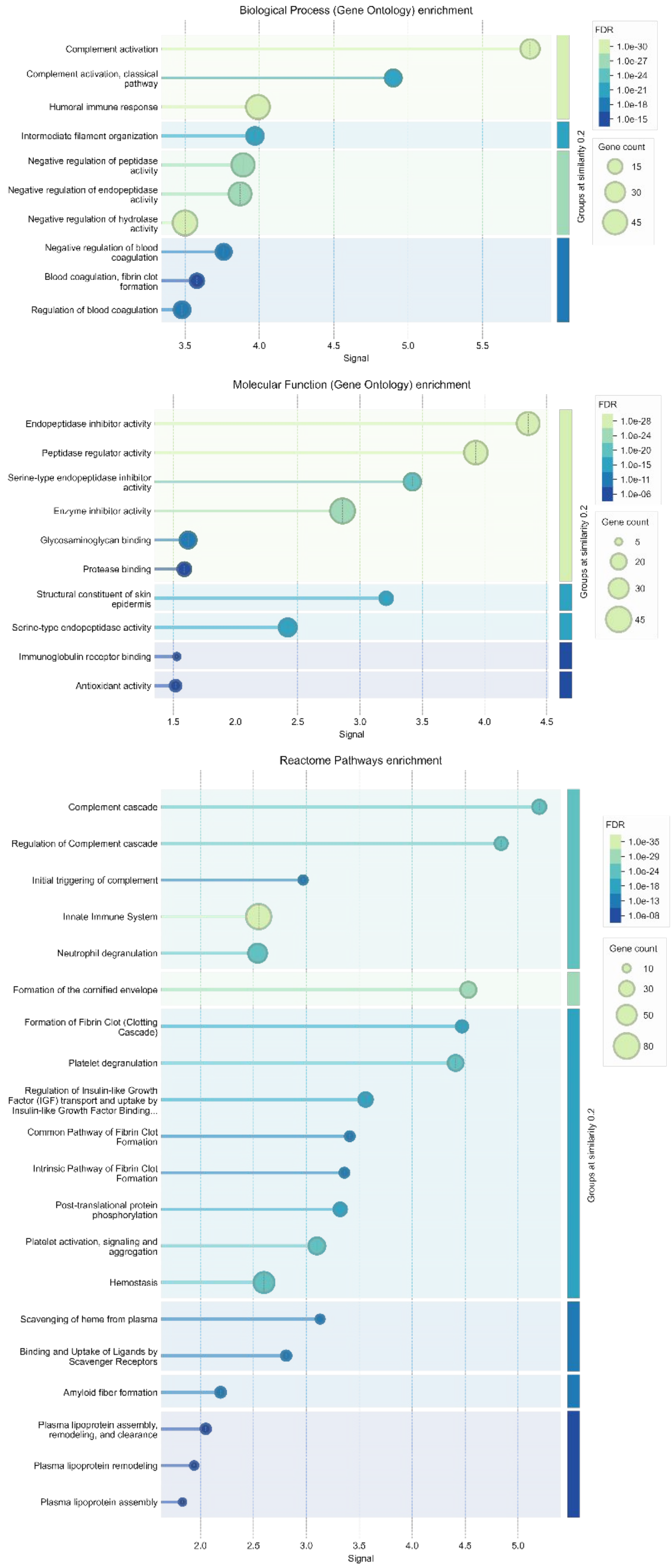

Figure S 3. PPI Network of proteins from plasma adsorbed on PEO/Zn surface.
